# Machine learning-based risk prediction model for sepsis development in patients with multidrug-resistant *Pseudomonas aeruginosa* infections: a multicenter retrospective cohort study

**DOI:** 10.3389/fcimb.2026.1792743

**Published:** 2026-04-10

**Authors:** Chang Li, Ting Shi, Guanyu Xiao, Yixin Zhang, Yuanyuan Wang, Yong Liang, Chaogui Tang, Ning Lin, Kai Wang

**Affiliations:** 1 Department of Medical Laboratory, The Affiliated Huai’an No. 1 People’s Hospital of Nanjing Medical University, Huai’an, Jiangsu, China; 2 Department of Hepatopancreatobiliary Surgery, The Affiliated Huai’an No. 1 People’s Hospital of Nanjing Medical University, Huai’an, Jiangsu, China; 3 Department of Clinical Laboratory, The Affiliated Huaian Hospital of Xuzhou Medical University, Huai’an, Jiangsu, China; 4 Department of Biostatistics, Center for Global Health, School of Public Health, Nanjing Medical University, Nanjing, Jiangsu, China; 5 Department of Rheumatology, The Affiliated Huai’an No. 1 People’s Hospital of Nanjing Medical University, Huai’an, Jiangsu, China

**Keywords:** clinical decision support, machine learning, multidrug-resistant *Pseudomonas aeruginosa*, risk prediction, sepsis, SHAP

## Abstract

**Background:**

Multidrug-resistant *Pseudomonas aeruginosa* (MDR-PA) infections present a critical healthcare challenge, often progressing to sepsis with high mortality. Current prediction tools lack specificity for drug-resistant organisms, hindering the early identification of high-risk patients. This study aimed to develop and validate an interpretable machine learning (ML) model to predict sepsis development in patients with MDR-PA infections.

**Methods:**

We conducted a multicenter retrospective study analyzing 2,001 patients with laboratory-confirmed MDR-PA infections from two major medical centers between January 2019 and May 2025. The derivation cohort included 1,182 patients, while 819 patients from an independent center served as the external validation cohort. Feature selection was performed using a hybrid approach combining LASSO regression and support vector machine-recursive feature elimination (SVM-RFE). Seven ML algorithms were evaluated, with model interpretability enhanced via SHapley Additive exPlanations (SHAP). A web-based calculator was subsequently developed to facilitate clinical implementation.

**Results:**

The sepsis incidence was approximately 7% across cohorts. Feature selection identified six key predictors: calcium level, chronic obstructive pulmonary disease (COPD), red blood cell distribution width-standard deviation (RDW-SD), intra-abdominal infection, invasive catheters, and prior antibiotic exposure. The Random Forest model demonstrated superior performance, achieving an AUC of 1.000 in the SMOTE-balanced training set, 0.837 in internal validation, and 0.816 in external validation. SHAP analysis highlighted COPD and calcium levels as the most significant contributors to sepsis risk.

**Conclusions:**

This study presents the first interpretable ML model specifically tailored for predicting sepsis onset in patients with MDR-PA infections. By addressing the limitations of general sepsis scores, our validated model and accompanying web-based tool provide clinicians with a precise, visualizable decision-support system to optimize early intervention strategies.

## 1 Introduction

Multidrug-resistant *Pseudomonas aeruginosa* (MDR-PA) infections represent a critical healthcare challenge, contributing significantly to antimicrobial resistance and poor clinical outcomes worldwide (Sati et al., 2025). Classified by the World Health Organization as a “critical priority” pathogen, MDR-PA demonstrates remarkable adaptability and intrinsic resistance mechanisms that complicate therapeutic management (Kunz Coyne et al., 2022). These infections are associated with prolonged hospitalization, increased healthcare costs, and substantially elevated mortality rates, particularly when complicated by sepsis (Vena et al., 2024). Prior studies have identified several key risk factors associated with the acquisition of MDR-PA infections, including prolonged hospitalization, prior exposure to broad-spectrum antibiotics (particularly carbapenems and fluoroquinolones), invasive mechanical ventilation, and immunocompromised states (Herrera et al., 2021; Montrucchio et al., 2024; Vena et al., 2024). These factors not only predispose patients to colonization but also create a vulnerable physiological environment that facilitates the transition from localized infection to systemic dysregulation.

Sepsis, operationally defined as life-threatening organ dysfunction secondary to a dysregulated host immune response to infection, represents the gravest complication of MDR-PA infections, with associated mortality rates surpassing 40% in the critically ill population (Singer et al., 2016; Hunter et al., 2025). The transition from localized MDR-PA infection to sepsis involves complex pathophysiological processes that remain incompletely understood, making early identification challenging for clinicians. However, existing general prognostic models often lack specificity for high-risk subgroups. Specifically, the SOFA score primarily reflects organ dysfunction rather than the specific pathophysiological burden of multidrug-resistant (MDR) infections, potentially leading to a lag in risk stratification for patients with MDR-PA (Prasad et al., 2020; Routsi et al., 2020). MDR-PA warrants distinct attention compared to other multidrug-resistant organisms (e.g., MRSA or ESBL-producing Enterobacteriaceae) due to its unique virulence mechanisms. *P. aeruginosa* possesses an extensive arsenal of virulence factors, such as type III secretion systems (T3SS), exotoxin A, and robust biofilm formation capabilities, which can induce rapid tissue necrosis and immune evasion, leading to a more fulminant progression to septic shock (Zhang et al., 2021; Tuon et al., 2022). Furthermore, the extremely limited therapeutic options for MDR-PA often result in delayed effective antimicrobial therapy, a critical determinant of mortality that differs significantly from infections caused by pathogens with wider antibiotic coverage. Consequently, relying solely on these general scores may delay the initiation of appropriate targeted therapies for patients with drug-resistant infections (Fernando et al., 2018; Serafim et al., 2018).

Therefore, there is an urgent need for more accurate and timely predictive tools, machine learning (ML) techniques have demonstrated remarkable potential in developing sophisticated prediction models for clinical outcomes, offering advantages over traditional statistical approaches in handling complex, nonlinear relationships among multiple variables (Kulkarni and Singh, 2023; Swanson et al., 2023). Several studies have shown ML algorithms’ utility in sepsis prediction, achieving superior performance compared to conventional scoring systems (Cilloniz et al., 2023). However, most existing models have been developed for general patient populations without specific consideration of drug-resistant bacterial infections, limiting their applicability to MDR-PA cases.

Despite clear clinical need, significant limitations exist in current research. Most studies focus on general sepsis prediction without considering pathogen-specific factors that may influence disease progression (Lauritsen et al., 2020). Additionally, many existing models lack external validation across different healthcare institutions, limiting generalizability and clinical adoption. Furthermore, while ML algorithms often achieve high predictive accuracy, the ‘black box’ nature of complex models has historically hindered clinical acceptance. Healthcare providers require not just accurate predictions but also transparent, interpretable insights to support evidence-based decision-making (Ghassemi et al., 2021). Therefore, developing a model that balances high performance with interpretability remains a critical unmet need.

Furthermore, previous research has often been constrained by small sample sizes, single-center designs, or limited variable selection, potentially compromising model robustness and clinical applicability (Sendak et al., 2020a, b). A critical need persists for well-structured, multicenter investigations that establish and validate interpretable ML models specifically optimized for sepsis prediction in MDR-PA infected populations.

To address these pivotal gaps, we developed and externally validated an interpretable ML framework for early sepsis prediction in patients with MDR-PA infections using a comprehensive multicenter cohort. Our approach incorporated sophisticated feature selection techniques that synergistically combined LASSO regression and SVM-RFE methodologies to identify the most clinically relevant predictive variables. Multiple ML algorithms were systematically evaluated to determine the optimal prediction model. To enhance clinical acceptance, we utilized the SHAP method for transparent model interpretability and developed a user-friendly web-based calculator for real-time clinical application, creating a robust, clinically actionable tool for early identification of high-risk patients.

## 2 Data and methods

### 2.1 Study cohort

We collected data from patients with multidrug-resistant *Pseudomonas aeruginosa* (MDR-PA) infections who were admitted to the Affiliated Huai’an No. 1 People’s Hospital of Nanjing Medical University and the Affiliated Huai’an Hospital of Xuzhou Medical University between January 2019 to May 2025. The inclusion criteria were as follows: (1) patients had laboratory-confirmed MDR-PA infection based on antimicrobial susceptibility testing; (2) patients were aged ≥ 18 years; and (3) patients had a minimum hospital stay of 48 hours. A total of 1266 patients from the Affiliated Huai’an No. 1 People’s Hospital of Nanjing Medical University and 845 patients from the Affiliated Huai’an Hospital of Xuzhou Medical University met the inclusion criteria. The exclusion criteria were as follows: (1) missing key laboratory parameters; (2) patients who died within 24 hours of admission due to causes unrelated to sepsis; (3) patients with pre-existing sepsis or septic shock at the time of MDR-PA infection diagnosis; (4) MDR-PA was isolated either prior to admission, within 48h of hospitalization, or > 72h post-discharge; (5) patients with concurrent infections caused by other drug-resistant organisms; (6) patients who were transferred to other facilities before outcome assessment; and (7) patients with colonization by MDR-PA. Patients diagnosed with MDR-PA infection within the first 48 hours of hospital admission were excluded to specifically focus on nosocomial (hospital-acquired) infections, which represent a distinct clinical entity with different resistance profiles compared to community-acquired cases. Furthermore, to ensure the model’s specificity for disease progression, we strictly excluded patients with MDR-PA colonization (defined as a positive culture without clinical signs of active infection according to CDC criteria) (Hurley, 2025). The inclusion of colonized patients would introduce significant heterogeneity, as the pathophysiological trajectory from colonization to sepsis differs fundamentally from the progression of active infection to sepsis. The study ultimately enrolled 1,182 patients from the Affiliated Huai’an No. 1 People’s Hospital of Nanjing Medical University for training and internal validation purposes, with an additional 819 patients from the Affiliated Huai’an Hospital of Xuzhou Medical University serving as the external validation cohort. This research protocol was executed in full compliance with Declaration of Helsinki standards and received ethical approval from both institutional review boards (Ethics approval numbers: KY-2024-355-01, HEYLL202569). Given the retrospective methodology and complete patient data anonymization, informed consent was waived by the ethics committees.

### 2.2 Clinical features and data processing

To ensure temporal consistency, Time Zero (t0) was defined as the time of sample collection for the first positive culture confirming MDR-PA infection. All baseline demographic and clinical predictor variables were collected within a 24-hour window preceding t0 to capture the patient’s physiological status at the onset of confirmed infection. The outcome observation window extended from t0 until hospital discharge or death. Sepsis events occurring prior to t0 were excluded to ensure the model predicts future risk rather than identifying concurrent conditions.

Study variables were systematically abstracted from patients’ hospitalization electronic medical records (EMRs), including baseline demographics, clinical presentations, therapeutic approaches, comorbid conditions, and initial laboratory measurements. Baseline patient demographics comprised gender and age. Clinical history and status assessments included prior antibiotic exposure (defined as the administration of any systemic antimicrobial agent [intravenous or oral] for at least 48 hours within the preceding 90 days) and immunosuppression (defined as the presence of active malignancy, human immunodeficiency virus infection (Hurley, 2025), neutropenia, history of solid organ or hematopoietic stem cell transplantation, or current receipt of chemotherapy, radiotherapy, or corticosteroids [≥ 20 mg/day prednisone equivalent for ≥ 2 weeks]). Therapeutic interventions documented comprised invasive catheters (including central venous catheters, arterial lines, and urinary catheters), endotracheal intubation, mechanical ventilatory assistance, ventilator liberation failure, vasoactive pharmaceutical support, continuous renal replacement therapy (CRRT), extracorporeal membrane oxygenation (ECMO), and anticoagulant therapy. Comorbidity information obtained included hyperlipidemia, liver abscess, cholecystitis, cholangitis, intra-abdominal infection, pneumonia, urinary tract infection, coronary heart disease, altered mental status, multiple organ dysfunction syndrome (MODS), cardiac arrest, coagulopathy, disseminated intravascular coagulation (DIC), CKD, heart failure, COPD, stroke, metabolic encephalopathy, hepatic encephalopathy, atrial fibrillation, ventricular fibrillation, hyperlactatemia, hypertension, diabetes mellitus, diabetes-related complications, acute liver injury, acute kidney injury, anemia, intracranial infection, acute respiratory distress syndrome (ARDS), and skull and brain injury. Laboratory indicators obtained included blood gas analysis parameters [Urea, oxygen saturation (SO_2_), partial pressure of oxygen (PO_2_), pH value (pH), partial pressure of carbon dioxide (PCO_2_), lactate (Lac), bicarbonate (HCO_3_^-^), base excess (BE), carbon dioxide (CO_2_)], complete blood count parameters [white blood cell count (WBC), red blood cell count (RBC), platelet count (PLT), hemoglobin (HGB), hematocrit (HCT), mean corpuscular volume (MCV), mean corpuscular hemoglobin (MCH), mean corpuscular hemoglobin concentration (MCHC), red cell distribution width-standard deviation (RDW-SD), red cell distribution width-coefficient of variation (RDW-CV), platelet distribution width (PDW), plateletcrit (PCT), mean platelet volume (MPV)], differential blood count [neutrophil percentage, neutrophil count, lymphocyte percentage, lymphocyte count, monocyte percentage, monocyte count, eosinophil percentage, eosinophil count, basophil percentage, basophil count], coagulation function parameters [prothrombin time (PT), international normalized ratio (INR), activated partial thromboplastin time (APTT), fibrinogen, D-dimer], liver function parameters [total protein (TP), albumin (ALB), alanine aminotransferase (ALT), aspartate aminotransferase (AST), alkaline phosphatase (ALP), gamma-glutamyl transferase (γ-GGT), total bilirubin (TBIL), direct bilirubin (DBIL), total bile acid (TBA), lactate dehydrogenase (LDH), hydroxybutyrate dehydrogenase (α-HBDH), cholinesterase (CHE), adenosine deaminase (ADA), alpha-fetoprotein (AFU), prealbumin (PA)], renal function parameters [creatinine, cystatin C (CYSC), uric acid (UA)], lipid metabolism parameters [total cholesterol (CHOL), triglycerides (TG), high-density lipoprotein cholesterol (HDL-C), low-density lipoprotein cholesterol (LDL-C), electrolyte parameters [sodium, potassium, calcium, magnesium, phosphate], inflammatory markers procalcitonin (PCT), cardiac markers [creatine kinase (CK), thrombin time (TT)]. Based on data preprocessing, the following inflammation-related composite indices were calculated: neutrophil-to-lymphocyte ratio (NLR) and platelet-to-lymphocyte ratio (PLR). To ensure temporal precedence and prevent data leakage, all predictor variables were strictly collected prior to the onset of sepsis. Specifically, baseline demographics, comorbidities, and laboratory parameters were derived from the record closest to t0 (within the 24-hour window preceding t0). Therapeutic interventions (e.g., antibiotic use, invasive procedures) were included only if they were administered before t0. (detailed variable descriptions and coding schemes are provided in **Supplementary Table 1**, **S2**).

### 2.3 Assessment of study outcomes

Sepsis identification in MDR-PA infected patients was conducted in accordance with the Third International Consensus Definitions for Sepsis and Septic Shock (Sepsis-3) diagnostic criteria. Sepsis was clinically defined as life-threatening organ dysfunction precipitated by dysregulated host responses to infectious processes, identified by Sequential Organ Failure Assessment (SOFA) score increments of ≥ 2 points from baseline, corresponding to hospital mortality greater than 10%, the specific diagnostic criteria included: (1) suspected or documented infection based on clinical presentation and/or laboratory evidence; and (2) acute change in total SOFA score ≥ 2 points consequent to the infection. It is important to note that the diagnosis of sepsis and the primary outcome were defined exclusively based on the SOFA score criteria. While the quick SOFA (qSOFA) score was calculated for rapid bedside screening and baseline characterization, it was not used as a diagnostic criterion for the study outcome (Singer et al., 2016).

### 2.4 Data preprocessing

First, variables with a missing rate exceeding 20% were excluded from the analysis to ensure data quality. For the remaining variables, missing values were addressed using the Multiple Imputation by Chained Equations (MICE) algorithm via the ‘mice’ R package. We employed predictive mean matching (PMM) for continuous variables and logistic regression (logreg) for binary variables to preserve the distributional characteristics of the data. A single imputed dataset was generated and used for the subsequent multivariate analysis.

Regarding outliers, continuous variables were screened using box plots, values exceeding 1.5 times the interquartile range (IQR) were winsorized (truncated to the boundary thresholds) rather than removed, preserving sample size while mitigating the impact of extremes. Finally, categorical variables were converted into numerical formats using one-hot encoding. Crucially, all preprocessing steps (imputation, winsorization, and encoding) were derived solely from the training set and then applied to the validation set to prevent data leakage.

### 2.5 Balancing data for enhanced predictive modeling

Of the 1,182 patients in the derivation cohort, data were randomly split into a training set (n = 827, 70%) and an internal validation set (n = 355, 30%). In the initial training set (n = 827), the incidence of sepsis was 7.01% (58 patients), indicating a significant class imbalance that could bias the model toward the majority class (non-sepsis) and compromise predictive accuracy.

To address this, we applied the Synthetic Minority Over-sampling Technique (SMOTE) exclusively to the training set. SMOTE generates synthetic minority-class samples by interpolating between a minority instance and its k-nearest minority neighbors, rather than simply duplicating existing observations. The application of SMOTE increased the minority class representation in our training dataset from 7.01% (58 cases) to 49.51% (754 cases), resulting in a balanced distribution. Crucially, the internal validation set (n = 355) and the external validation cohort (n = 819) were kept in their original imbalanced state containing only real patient data to ensure an unbiased evaluation of the model’s performance in real-world clinical scenarios.

### 2.6 Selection of variables

We deployed a composite variable selection architecture merging least absolute shrinkage and selection operator (LASSO) methodology with support vector machine-recursive feature elimination (SVM-RFE) algorithms to maximize predictive precision and strengthen algorithmic resilience. First, LASSO regression was applied for initial feature selection by adding a penalty term that shrinks coefficients toward zero, effectively performing automatic variable selection (Hsu et al., 2024). The optimal penalty parameter (λ) was determined through 10-fold cross-validation. Subsequently, RFE was applied to the LASSO-selected features. SVM-RFE works by recursively fitting the model, ranking features by importance, and eliminating the least important features until the optimal number of features is reached (Rani and Babu, 2024). We applied iterative 10-fold cross-validation across 10 cycles to comprehensively validate model performance and demonstrate consistent predictive reliability. This sequential combination has been validated in prior research to outperform single methods by effectively filtering noise while preserving informative features (Shi et al., 2021). Multicollinearity among candidate predictors was assessed using the variance inflation factor (VIF), all variables showed acceptable collinearity (all VIFs < 5), and therefore no predictors were excluded on the basis of multicollinearity.

### 2.7 Model development and validation

Seven algorithmic approaches were applied to predict sepsis onset risk in patients harboring MDR-PA infections: logistic regression (LR), decision tree (DT), random forest (RF), extreme gradient boosting (XGBoost), support vector machine (SVM), K-nearest neighbor (KNN), and Light Gradient Boosting Machine (LightGBM). Algorithmic refinement utilized comprehensive parameter optimization applied to the curated feature set, integrating repeated validation protocols (10 × 10-fold cross-validation) with systematic hyperparameter space exploration. For the LR model, we applied L2 regularization to prevent overfitting. The DT model was constructed with pruning parameters to avoid overfitting while maintaining interpretability. The RF model utilized bootstrap aggregating with multiple decision trees to improve prediction stability and accuracy. XGBoost employed gradient boosting framework with advanced regularization techniques. The SVM model used radial basis function kernel for handling non-linear relationships. KNN was implemented with optimal neighbor selection based on cross-validation performance. LightGBM was configured with gradient-based one-side sampling and exclusive feature bundling for efficient training. Finally, the models were refitted on the training set with the optimal feature subset and the final hyperparameters based on 10 rounds of 10-fold internal cross-validation. All models were developed using standardized data preprocessing procedures to ensure fair comparison and optimal performance across different algorithms.

### 2.8 Model performance comparison

The reliability and performance of the models were comprehensively evaluated using multiple well-established metrics. The discriminatory performance of the model was evaluated using the area under the receiver operating characteristic curve (AUC). Additionally, model performance was assessed using standard classification metrics, including sensitivity, specificity, positive predictive value (PPV), negative predictive value (NPV), accuracy, F1 score, and the Brier score. Model calibration was assessed via Hosmer-Lemeshow analysis to determine predicted-observed probability alignment. Calibration visualization through probability plots revealed prediction accuracy across different threshold ranges, demonstrating algorithmic consistency. Decision curve analysis (DCA) quantified the clinical effectiveness and net therapeutic advantage of each algorithm across multiple decision thresholds, providing comprehensive evaluation of their practical healthcare implementation potential. The optimal prediction model was selected based on the comprehensive evaluation of all performance metrics in both the training and external validation sets, ensuring robust model selection that balances discrimination, calibration, and clinical utility.

### 2.9 Model explanation

Machine learning model interpretability faces challenges due to algorithmic complexity. We employed SHapley Additive exPlanations (SHAP), a game theory-founded method that assesses feature impact and demystifies computational decision-making frameworks. The SHAP framework provides both global explanations revealing overall feature importance patterns and local explanations detailing individual patient predictions. By calculating each feature’s marginal contribution to prediction outcomes, SHAP enhances model transparency and clinical acceptability, enabling healthcare practitioners to understand the reasoning behind predictions and fostering confidence in ML-assisted decision-making (Hsu et al., 2023).

### 2.10 Network calculator

To facilitate clinical implementation, the final prediction model was integrated into a user-friendly Shiny web platform. By inputting relevant clinical and laboratory parameters, the application provides real-time probability assessments for sepsis development in patients with MDR-PA infections. This web-based calculator enables clinicians to obtain immediate risk stratification, supporting evidence-based decision-making and timely clinical interventions.

### 2.11 Statistics

Algorithmic development employed R computational platform (version 4.3.1) with “caret” package integration (6.0.94) for standardized machine learning implementation. Seven algorithms were operationalized through train function methodology: generalized linear modeling (glm), recursive partitioning (rpart), ensemble random forest (ranger), extreme gradient boosting (xgbTree), radial support vector machines (svmRadial), k-nearest neighbor classification (knn), and lightweight gradient boosting (lightgbm). Performance discrimination assessment utilized receiver operating characteristic curve analysis, producing AUC values with bootstrap-derived 95% confidence intervals (1000 replications). Calibration evaluation incorporated Brier score calculation (0–1 spectrum), where minimal values indicate optimal prediction-observation agreement. Hosmer-Lemeshow statistical testing provided additional calibration assessment, with P-values exceeding 0.05 confirming satisfactory model fit. Decision curve methodology quantified clinical utility through net benefit analysis across probability threshold ranges.

Statistical analyses were performed according to data type and distribution. First, for continuous variables, normality was assessed using the Shapiro-Wilk test (or visual inspection of histograms). Normally distributed data were expressed as mean ± standard deviation (SD) and compared using Student’s t-test. Conversely, non-normally distributed data were presented as median with interquartile range (IQR) and analyzed using the Mann-Whitney U test (for two groups). Second, categorical variables were summarized as frequencies and percentages (n, %), with group comparisons performed using Pearson’s chi-square test or Fisher’s exact test, as appropriate. All statistical tests were two-sided, and a P-value < 0.05 was considered statistically significant.

## 3 Results

### 3.1 Characteristics of patients with MDR-PA infection in a retrospective study

**Figure 1:**
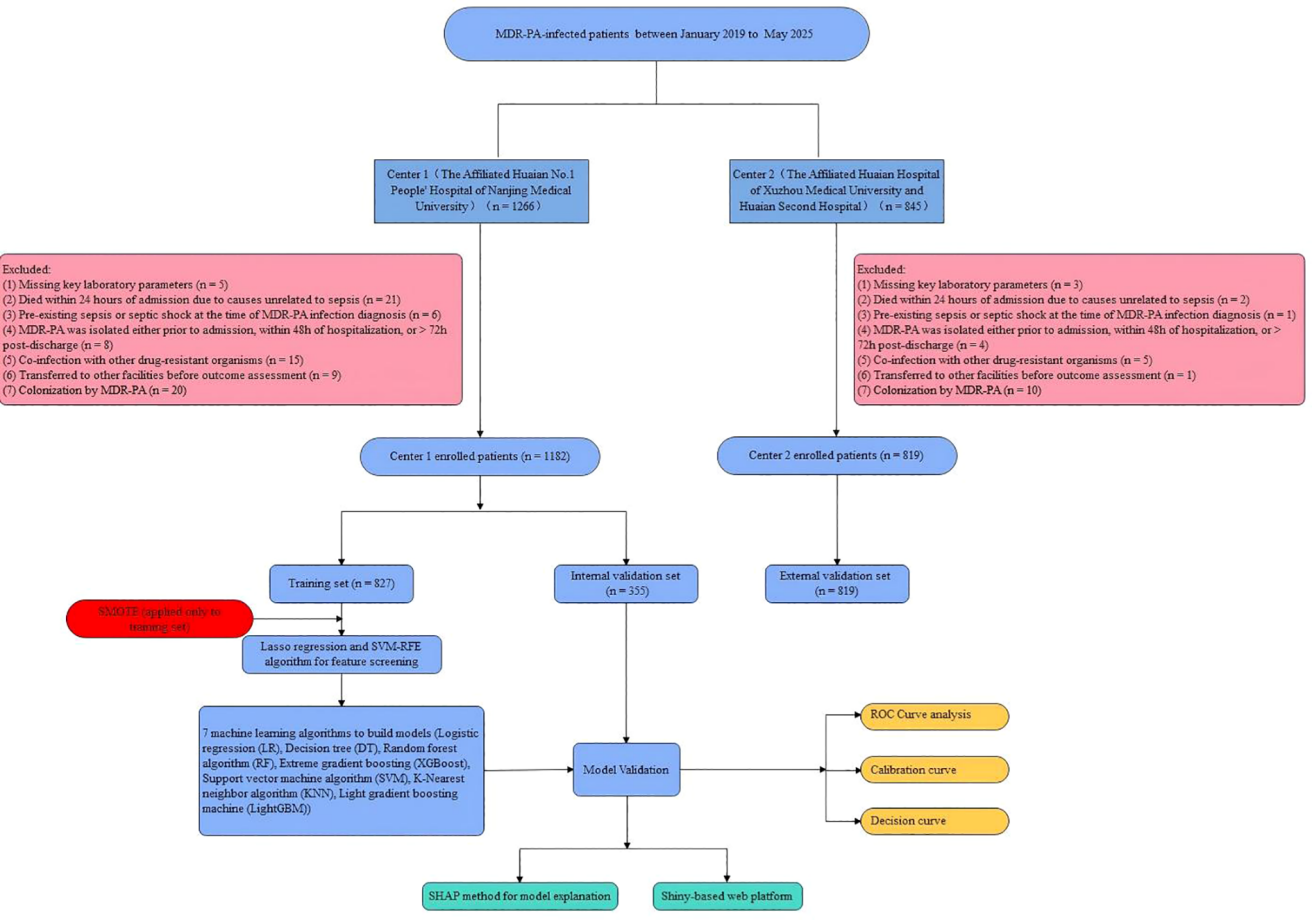
Study flowchart. MDR-PA, multidrug-resistant *Pseudomonas aeruginosa*; LASSO, least absolute shrinkage and selection operator; SVM-RFE, support vector machine-recursive feature elimination; LR, logistic regression; DT, decision tree; RF, random forest; XGBoost, extreme gradient boosting; SVM, support vector machine; KNN, k-nearest neighbor; LightGBM, light gradient boosting machine; SHAP, SHapley Additive exPlanations; ROC, receiver operating characteristic.

The study population comprised patients with MDR-PA infections at The Affiliated Huai’an No.1 People’s Hospital of Nanjing Medical University between January 2019 to May 2025. From an initial cohort of 1,266 patients, 1,182 met eligibility criteria and comprised the derivation cohort. The derivation cohort was randomly divided into a training set (n = 827) and internal validation set (n = 355) at a 7:3 ratio. Baseline characteristics showed no significant differences between groups, confirming their comparability. For external validation, an independent cohort of 819 patients was recruited from the Affiliated Huai’an Hospital of Xuzhou Medical University during the same period using identical criteria, this external cohort assessed model generalizability and robustness across different healthcare institutions. The detailed study design, including the definition of prediction windows and outcome assessment, is illustrated in **Supplementary Figure 1** and **Figure 1**.

### 3.2 Incidence of sepsis after MDR-PA infection

The overall sepsis incidence following MDR-PA infection was 7.02% (83/1,182) in the derivation cohort, with comparable rates in the training [7.01% (58/827)] and internal validation [7.04% (25/355)] subsets. To address this imbalance during model training, SMOTE was applied, resulting in a balanced distribution between sepsis and non-sepsis cases in the training set (**Supplementary Table 3**). In the external validation cohort (n = 819), the sepsis incidence was 7.33% (60/819), demonstrating consistency across healthcare institutions.

Comparison of baseline characteristics between the derivation and external validation cohorts revealed similarities and differences (**Table 1**). A comprehensive analysis of baseline characteristics revealed significant differences between groups in 19 variables, including age, HCT, PDW, lymphocyte count, basophils count, eosinophil percentage and absolute number, PCT, SO_2_, phosphate, CHOL, hyperlipidemia, hypertension, heart failure, cholecystitis, cholangitis, stroke, coagulopathy and magnesium (all P < 0.05).

**Table 1:** Comparison of demographic characteristics and clinical characteristics between two centers.

Characteristic	Total (N = 2001)	Center 1 (N = 1182)	Center 2 (N = 819)	*P*-value
Age (year), Median (IQR)	67.00 [56.00; 77.00]	69.00 [57.00; 78.00]	67.00 [56.00; 75.00]	**0.042**
Gender, *n* (%)				0.151
Female	516 (25.79%)	291 (24.62%)	225 (27.47%)	
Male	1485 (74.21%)	891 (75.38%)	594 (72.53%)	
Laboratory results				
WBC count (10^9^/L), Median (IQR)	8.72 [6.42; 12.37]	8.64 [6.32; 12.26]	8.87 [6.54; 12.48]	0.119
HGB (g/L), Median (IQR)	110.00 [93.00; 125.00]	109.00 [93.00; 125.00]	111.00 [92.00; 126.00]	0.221
RBC count (10^12^/L), Median (IQR)	3.69 [3.14; 4.17]	3.69 [3.13; 4.18]	3.70 [3.16; 4.16]	0.657
HCT (%), Median (IQR)	34.00 [29.00; 38.40]	34.00 [29.00; 38.48]	34.15 [29.15; 38.65]	**0.030**
RDW-SD (fL), Median (IQR)	46.20 [43.20; 50.40]	46.20 [43.30; 50.40]	46.20 [43.15; 50.20]	0.739
RDW-CV (%), Median (IQR)	13.90 [13.10; 15.00]	13.90 [13.10; 15.00]	14.00 [13.00; 15.10]	0.961
MCV (fL), Median (IQR)	92.60 [89.10; 96.10]	92.60 [89.20; 96.20]	92.40 [89.00; 95.90]	0.341
MCHC (g/L), Median (IQR)	325.00 [315.00; 333.00]	324.00 [315.00; 331.00]	325.00 [314.00; 334.00]	0.886
MCH (pg), Median (IQR)	29.80 [28.80; 31.20]	29.90 [28.80; 31.20]	29.80 [28.70; 31.10]	0.400
PLT count (10^9^/L), Median (IQR)	202.00 [148.00; 276.00]	202.00 [149.00; 276.00]	203.00 [148.00; 278.50]	0.471
PDW (fL), Median (IQR)	11.90 [10.10; 15.50]	11.90 [10.12; 15.47]	11.70 [10.00; 15.30]	**0.012**
MPV (fL), Median (IQR)	10.00 [9.20; 11.00]	10.10 [9.30; 11.10]	10.00 [9.20; 10.90]	0.674
Neutrophils percentage (%), Median (IQR)	80.10 [70.30; 88.00]	79.90 [69.82; 87.90]	80.30 [70.90; 88.00]	0.419
Neutrophils count (10^9^/L), Median (IQR)	6.76 [4.52; 10.43]	6.76 [4.45; 10.40]	6.79 [4.54; 10.51]	0.724
Monocytes percentage (%), Median (IQR)	6.00 [4.40; 7.70]	6.00 [4.40; 7.80]	5.90 [4.20; 7.60]	0.432
Monocytes count (10^9^/L), Median (IQR)	0.51 [0.36; 0.71]	0.51 [0.36; 0.71]	0.50 [0.35; 0.71]	0.502
Lymphocytes percentage (%), Median (IQR)	11.70 [6.30; 20.00]	11.70 [6.32; 20.30]	11.40 [6.20; 19.75]	0.467
Lymphocytes count (10^9^/L), Median (IQR)	1.01 [0.64; 1.49]	1.04 [0.66; 1.52]	0.98 [0.62; 1.48]	**0.025**
Basophils percentage (%), Median (IQR)	0.20 [0.10; 0.30]	0.20 [0.10; 0.30]	0.20 [0.11; 0.31]	0.146
Basophils count (10^9^/L), Median (IQR)	0.02 [0.01; 0.03]	0.02 [0.01; 0.03]	0.03 [0.02; 0.04]	**0.045**
Eosinophils percentage (%), Median (IQR)	0.50 [0.10; 1.40]	0.50 [0.10; 1.43]	0.50 [0.10; 1.20]	**0.037**
Eosinophils count (10^9^/L), Median (IQR)	0.06 [0.01; 0.15]	0.06 [0.01; 0.16]	0.05 [0.01; 0.14]	**0.035**
NLR, Median (IQR)	6.72 [3.46; 13.63]	6.70 [3.42; 13.42]	6.90 [3.63; 14.07]	0.455
PLR, Median (IQR)	73.40 [47.97; 117.52]	73.38 [47.88; 116.11]	75.33 [48.24; 119.85]	0.458
PCT, Median (IQR)	0.22 [0.16; 0.27]	0.23 [0.16; 0.27]	0.20 [0.15; 0.27]	**0.045**
Urea (mmol/L), Median (IQR)	5.95 [4.42; 8.10]	5.95 [4.43; 8.23]	5.97 [4.41; 7.96]	0.542
Creatinine (μmol/L), Median (IQR)	55.25 [44.00; 76.30]	55.25 [44.00; 75.95]	55.35 [44.00; 77.05]	0.965
UA (μmol/L), Median (IQR)	211.00 [156.00; 283.00]	211.00 [156.00; 283.00]	208.00 [152.00; 281.00]	0.283
CYSC (mg/L), Median (IQR)	1.04 [0.85; 1.31]	1.04 [0.85; 1.32]	1.03 [0.83; 1.30]	0.470
Serum CO_2_ (mmol/L), Median (IQR)	25.30 [24.80; 26.00]	25.30 [24.80; 26.00]	25.50 [24.30; 27.00]	0.830
TBIL (μmol/L), Median (IQR)	9.21 [6.42; 13.70]	9.20 [6.40; 13.70]	9.20 [6.50; 13.80]	0.700
DBIL (μmol/L), Median (IQR)	4.50 [3.21; 6.50]	4.50 [3.20; 6.40]	4.52 [3.20; 6.60]	0.653
AST (U/L), Median (IQR)	22.50 [16.70; 33.40]	22.40 [16.50; 33.30]	22.70 [16.90; 33.85]	0.082
ALT (U/L), Median (IQR)	19.40 [12.80; 32.00]	19.40 [12.70; 31.80]	19.60 [12.90; 32.10]	0.679
TBA (U/L), Median (IQR)	3.30 [2.10; 5.40]	3.30 [2.10; 5.57]	3.25 [2.05; 5.30]	0.522
TP (g/L), Mean (SD)	61.00 [55.50; 65.80]	60.90 [55.52; 65.97]	61.20 [55.55; 66.70]	0.648
ALB (g/L), Mean (SD)	35.00 [31.10; 38.40]	35.00 [31.20; 38.40]	34.90 [31.00; 38.20]	0.250
AFU (U/L), Median (IQR)	18.10 [15.80; 21.30]	18.20 [15.80; 21.40]	18.00 [15.90; 21.20]	0.888
CHE (U/L), Median (IQR)	4781.00 [3623.00; 6157.00]	4811.50 [3642.00; 6184.75]	4710.50 [3603.50; 6117.50]	0.573
PA (mg/dL), Median (IQR)	154.00 [119.20; 193.60]	153.00 [118.30; 194.88]	155.00 [120.15; 196.35]	0.469
α-HBDH (U/L), Median (IQR)	161.00 [147.00; 175.00]	161.00 [148.00; 175.00]	160.00 [145.50; 173.00]	0.931
LDH (U/L), Median (IQR)	204.00 [191.00; 226.00]	205.00 [192.25; 228.75]	203.00 [189.00; 223.00]	0.476
ALP (U/L), Median (IQR)	85.00 [67.00; 107.00]	85.00 [67.00; 106.00]	84.00 [65.00; 107.00]	0.845
γ-GGT (U/L), Median (IQR)	29.20 [18.10; 54.00]	29.00 [17.00; 54.00]	29.40 [18.10; 54.60]	0.577
ADA (U/L), Median (IQR)	12.00 [10.00; 14.10]	12.00 [10.00; 14.00]	12.10 [9.80; 14.10]	0.966
SO_2_ (%), Median (IQR)	97.60 [97.30; 97.85]	97.50 [97.20; 97.70]	97.60 [97.50; 97.95]	**0.037**
pH, Median (IQR)	7.42 [7.42; 7.42]	7.42 [7.42; 7.43]	7.42 [7.42; 7.42]	0.659
HCO_3_^-^(mmol/L), Median (IQR)	27.40 [26.90; 27.80]	27.40 [26.90; 27.98]	27.50 [27.20; 27.70]	0.869
BE (mmol/L), Median (IQR)	3.15 [2.60; 3.60]	3.15 [2.60; 3.70]	3.17 [2.65; 3.62]	0.876
PO_2_ (mmHg), Median (IQR)	90.60 [88.20; 95.00]	90.60 [87.45; 94.47]	90.80 [89.55; 96.45]	0.449
PCO_2_ (mmHg), Median (IQR)	41.80 [41.20; 42.50]	41.80 [41.12; 42.68]	42.00 [41.20; 42.80]	0.678
AG (mmHg), Median (IQR)	10.80 [10.30; 11.10]	10.70 [10.30; 11.10]	10.80 [10.40; 11.30]	0.194
Lac (mmol/L), Median (IQR)	1.70 [1.61; 1.70]	1.70 [1.60; 1.70]	1.71 [1.61; 1.73]	0.099
TG (mmol/L), Median (IQR)	1.09 [0.81; 1.56]	1.09 [0.81; 1.53]	1.10 [0.80; 1.58]	0.768
CHOL (mmol/L), Median (IQR)	3.41 [2.73; 4.10]	3.51 [2.85; 4.11]	3.41 [2.71; 4.08]	**0.044**
LDL (mmol/L), Median (IQR)	1.80 [1.80; 1.82]	1.80 [1.80; 1.81]	1.82 [1.78; 1.82]	0.679
HDL (mmol/L), Median (IQR)	0.94 [0.94; 0.94]	0.94 [0.94; 0.94]	0.96 [0.94; 1.01]	0.735
TT (s), Median (IQR)	17.30 [16.00; 18.00]	17.00 [16.00; 18.10]	17.50 [15.90; 18.20]	0.280
PT (s), Median (IQR)	13.50 [12.40; 14.70]	13.40 [12.40; 14.60]	13.60 [12.30; 14.90]	0.720
INR, Median (IQR)	1.12 [1.03; 1.19]	1.10 [1.03; 1.19]	1.12 [1.03; 1.21]	0.808
APTT (s), Median (IQR)	32.90 [28.80; 38.40]	32.90 [28.90; 38.30]	33.00 [28.70; 38.65]	0.794
D-dimer (mg/L), Median (IQR)	1.83 [0.91; 3.71]	1.85 [0.91; 3.75]	1.83 [0.92; 3.67]	0.957
Fibrinogen (g/L), Median (IQR)	4.19 [3.21; 5.29]	4.19 [3.23; 5.24]	4.21 [3.18; 5.35]	0.862
Phosphate (mmol/L), Median (IQR)	1.04 [0.86; 1.22]	1.04 [0.86; 1.21]	1.06 [0.88; 1.25]	**0.041**
Sodium (mmol/L), Median (IQR)	139.50 [136.60; 142.00]	139.30 [136.60; 141.90]	140.10 [136.50; 142.00]	0.876
Magnesium (mmol/L), Median (IQR)	0.89 [0.82; 0.94]	0.88 [0.81; 0.94]	0.91 [0.83; 0.96]	**0.035**
Potassium (mmol/L), Median (IQR)	4.04 [3.76; 4.32]	4.05 [3.77; 4.33]	4.03 [3.75; 4.31]	0.287
Calcium (mmol/L), Median (IQR)	2.16 [2.07; 2.25]	2.17 [2.07; 2.26]	2.15 [2.05; 2.23]	0.654
CK (U/L), Median (IQR)	52.00 [29.00; 106.00]	50.00 [29.25; 100.75]	54.00 [29.00; 110.00]	0.864
Treatment, *n* (%)				
Endotracheal intubation, *n* (%)				0.100
No	1526 (76.26%)	886 (74.96%)	640 (78.14%)	
Yes	475 (23.74%)	296 (25.04%)	179 (21.86%)	
Mechanical ventilation, *n* (%)				0.126
No	1225 (61.22%)	740 (62.61%)	485 (59.22%)	
Yes	776 (38.78%)	442 (37.39%)	334 (40.78%)	
Ventilator weaning failure, *n* (%)				0.071
No	1946 (97.25%)	1156 (97.80%)	790 (96.46%)	
Yes	55 (2.75%)	26 (2.20%)	29 (3.54%)	
Vasoactive medications, *n* (%)				0.075
No	1821 (91.00%)	1075 (90.95%)	763 (93.16%)	
Yes	180 (9.00%)	107 (9.05%)	56 (6.84%)	
CRRT use, *n* (%)				0.052
No	1886 (94.25%)	1113 (94.16%)	787 (96.09%)	
Yes	115 (5.75%)	69 (5.84%)	32 (3.91%)	
ECMO use, *n* (%)				0.082
No	1994 (99.65%)	1178 (99.66%)	811 (99.02%)	
Yes	7 (0.35%)	4 (0.34%)	8 (0.98%)	
Anticoagulant usage, *n* (%)				0.060
No	1513 (75.61%)	876 (74.11%)	637 (77.78%)	
Yes	488 (24.39%)	306 (25.89%)	182 (22.22%)	
Prior antibiotic exposure, *n* (%)				0.349
No	1895 (94.70)	1124 (95.09)	771 (94.14)	
Yes	106 (5.30)	58 (4.91)	48 (5.86)	
Immunosuppression, *n* (%)				1.000
No	1998 (99.85)	1180 (99.83)	818 (99.88)	
Yes	3 (0.15)	2 (0.17)	1 (0.12)	
Invasive catheters, *n* (%)				0.061
No	1883 (94.10)	1122 (94.92)	761 (92.92)	
Yes	118 (5.90)	60 (5.08)	58 (7.08)	
Comorbidity, *n* (%)				
Hyperlipidemia, *n* (%)				**< 0.001**
No	1970 (98.45%)	1164 (98.48%)	754 (92.06%)	
Yes	31 (1.55%)	18 (1.52%)	65 (7.94%)	
Hyperlactatemia, *n* (%)				0.058
No	1980 (98.95%)	1170 (98.98%)	802 (97.92%)	
Yes	21 (1.05%)	12 (1.02%)	17 (2.08%)	
Hypertension, *n* (%)				**0.047**
No	1017 (50.82%)	589 (49.83%)	445 (54.33%)	
Yes	984 (49.18%)	593 (50.17%)	374 (45.67%)	
Diabetes mellitus, *n* (%)				0.098
No	1551 (77.51%)	901 (76.23%)	650 (79.37%)	
Yes	450 (22.49%)	281 (23.77%)	169 (20.63%)	
Diabetes-related complications, *n* (%)				0.075
No	1952 (97.55%)	1147 (97.04%)	805 (98.29%)	
Yes	49 (2.45%)	35 (2.96%)	14 (1.71%)	
Coronary heart disease, *n* (%)				0.311
No	1732 (86.56%)	1015 (85.87%)	717 (87.55%)	
Yes	269 (13.44%)	167 (14.13%)	102 (12.45%)	
Atrial fibrillation, *n* (%)				0.063
No	1840 (91.95%)	1098 (92.89%)	742 (90.60%)	
Yes	161 (8.05%)	84 (7.11%)	77 (9.40%)	
History of CKD, *n* (%)				0.102
No	1959 (97.90%)	1157 (97.88%)	792 (96.70%)	
Yes	42 (2.10%)	25 (2.12%)	27 (3.30%)	
Heart failure, *n* (%)				**0.010**
No	1811 (90.50%)	1066 (90.19%)	708 (86.45%)	
Yes	190 (9.50%)	116 (9.81%)	111 (13.55%)	
Anemia, *n* (%)				0.085
No	1605 (80.21%)	933 (78.93%)	672 (82.05%)	
Yes	396 (19.79%)	249 (21.07%)	147 (17.95%)	
History of COPD, *n* (%)				0.198
No	1460 (72.96%)	875 (74.03%)	585 (71.43%)	
Yes	541 (27.04%)	307 (25.97%)	234 (28.57%)	
**Infection site, *n* (%)**				
Liver abscess, *n* (%)				0.070
No	1997 (99.80%)	1180 (99.83%)	812 (99.15%)	
Yes	4 (0.20%)	2 (0.17%)	7 (0.85%)	
Cholecystitis, *n* (%)				**0.003**
No	1934 (96.65%)	1154 (97.63%)	780 (95.24%)	
Yes	67 (3.35%)	28 (2.37%)	39 (4.76%)	
Cholangitis, *n* (%)				**0.007**
No	1981 (99.00%)	1172 (99.15%)	800 (97.68%)	
Yes	20 (1.00%)	10 (0.85%)	19 (2.32%)	
Intra-abdominal infection, *n* (%)				0.151
No	1967 (98.30%)	1166 (98.65%)	801 (97.80%)	
Yes	34 (1.70%)	16 (1.35%)	18 (2.20%)	
Pneumonia, *n* (%)				0.062
No	521 (26.04%)	306 (25.89%)	243 (29.67%)	
Yes	1480 (73.96%)	876 (74.11%)	576 (70.33%)	
Urinary tract infection, *n* (%)				0.056
No	1910 (95.45%)	1137 (96.19%)	773 (94.38%)	
Yes	91 (4.55%)	45 (3.81%)	46 (5.62%)	
Intracranial infection, *n* (%)				0.053
No	1912 (95.55%)	1130 (95.60%)	767 (93.65%)	
Yes	89 (4.45%)	52 (4.40%)	52 (6.35%)	
Acute organ injury, *n* (%)				
Acute liver injury, *n* (%)				0.295
No	1739 (86.91%)	1035 (87.56%)	704 (85.96%)	
Yes	262 (13.09%)	147 (12.44%)	115 (14.04%)	
Acute kidney injury, *n* (%)				0.061
No	1928 (96.35%)	1139 (96.36%)	775 (94.63%)	
Yes	73 (3.65%)	43 (3.64%)	44 (5.37%)	
Skull and brain injury, *n* (%)				0.082
No	1737 (86.81%)	1039 (87.90%)	698 (85.23%)	
Yes	264 (13.19%)	143 (12.10%)	121 (14.77%)	
MODS, *n* (%)				0.211
No	1990 (99.45%)	1175 (99.41%)	810 (98.90%)	
Yes	11 (0.55%)	7 (0.59%)	9 (1.10%)	
Stroke, *n* (%)				**0.049**
No	1423 (71.11%)	836 (70.73%)	612 (74.73%)	
Yes	578 (28.89%)	346 (29.27%)	207 (25.27%)	
Cardiac arrest, *n* (%)				0.065
No	1959 (97.90%)	1163 (98.39%)	796 (97.19%)	
Yes	42 (2.10%)	19 (1.61%)	23 (2.81%)	
Altered mental status, *n* (%)				0.089
No	1702 (85.06%)	1017 (86.04%)	685 (83.64%)	
Yes	299 (14.94%)	165 (13.96%)	134 (16.36%)	
Metabolic encephalopathy, *n* (%)				0.082
No	1995 (99.70%)	1178 (99.66%)	811 (99.02%)	
Yes	6 (0.30%)	4 (0.34%)	8 (0.98%)	
Hepatic encephalopathy, *n* (%)				0.070
No	1997 (99.80%)	1180 (99.83%)	813 (99.27%)	
Yes	4 (0.20%)	2 (0.17%)	6 (0.73%)	
Ventricular fibrillation, *n* (%)				0.274
No	1995 (99.70%)	1179 (99.75%)	819 (100.00%)	
Yes	6 (0.30%)	3 (0.25%)	0 (0.00%)	
Complication, n (%)				
Coagulopathy, *n* (%)				**0.006**
No	1903 (95.10%)	1123 (95.01%)	798 (97.44%)	
Yes	98 (4.90%)	59 (4.99%)	21 (2.56%)	
DIC, *n* (%)				0.152
No	1993 (99.60%)	1175 (99.41%)	818 (99.88%)	
Yes	8 (0.40%)	7 (0.59%)	1 (0.12%)	
ARDS, *n* (%)				0.083
No	1148 (57.37%)	697 (58.97%)	451 (55.07%)	
Yes	853 (42.63%)	485 (41.03%)	368 (44.93%)	

Bold values indicate statistical significance (P < 0.05).

In contrast, the majority of assessed variables showed no significant inter-center differences. These included gender distribution and extensive laboratory profiles encompassing complete blood count parameters, inflammatory markers (NLR, PLR), renal and liver function indicators, and arterial blood gas analysis. Crucially, key clinical risk factors and interventions-specifically prior antibiotic exposure, immunosuppression, invasive catheters, mechanical ventilation, vasoactive medications, and renal replacement therapy-demonstrated similar distributions between the two centers (all P > 0.05). This consistency across a wide range of baseline characteristics supports the validity of pooling data or cross-center validation, despite the distinct institutional settings.

Compared to non-sepsis patients, the sepsis group exhibited significantly higher rates of prior antibiotic exposure and invasive catheter usage, as well as increased respiratory support requirements (endotracheal intubation, mechanical ventilation) and ventilator weaning failure. These patients also demonstrated elevated needs for vasoactive medications and CRRT, alongside a higher prevalence of CKD and COPD history. Furthermore, the sepsis group showed higher frequencies of secondary infections, MODS, ARDS, and coagulopathy. Laboratory findings revealed enhanced inflammatory responses with elevated neutrophil percentages, NLR, and PCT, while lymphocyte, monocyte, and eosinophil counts were reduced alongside decreased platelets and increased RDW-SD. Organ dysfunction was characterized by elevated urea, creatinine, TBIL, DBIL, prolonged PT, increased INR, D-dimer, and CK levels, with reduced TP, CHE, lipid profiles, phosphate, and calcium concentrations. These results demonstrate sepsis-associated multiorgan failure, severe systemic inflammation, and metabolic disturbances requiring intensive management (**Table 2**).

**Table 2:** Comparison of demographic characteristics and clinical characteristics between non-sepsis and sepsis patients in the training cohort.

Characteristic	Total, N = 827	Non-Sepsis, N = 769	Sepsis, N = 58	*P*-value
Age (year), Median (IQR)	68.00 (57.00, 77.00)	68.00 (57.00, 77.00)	69 (57.00, 75.00)	0.941
Gender, *n* (%)				0.229
Female	196 (23.70)	178 (23.15)	18 (31.03)	
Male	631 (76.30)	591 (76.85)	40 (68.97)	
Laboratory results				
WBC count (10^9^/L), Median (IQR)	8.76 (6.44, 12.64)	8.74 (6.47, 12.48)	9.30 (6.17, 14.04)	0.668
HGB (g/L), Median (IQR)	109.00 (93.00, 125.00)	109.00 (93.00, 125.00)	101.50 (86.50, 120.00)	0.078
RBC count(10^12^/L), Mean(SD)	3.67 ± 0.80	3.68 ± 0.79	3.48 ± 0.83	0.081
HCT (%), Mean(SD)	33.87 ± 7.21	34.00 ± 7.19	32.18 ± 7.23	0.069
RDW-SD(fL), Median (IQR)	46.20 (43.30, 50.10)	46.10 (43.10, 50.10)	47.00 (44.73, 52.10)	**0.023**
RDW-CV(%), Median (IQR)	13.80 (13.00, 15.00)	13.80 (13.00, 14.90)	14.15 (13.33, 15.35)	0.069
MCV(fL), Median (IQR)	92.60 (89.20, 96.10)	92.60 (89.20, 96.10)	93.00 (90.05, 97.15)	0.241
MCHC(g/L), Median (IQR)	324.00 (315.50, 331.50)	324.00 (315.00, 331.00)	326.00 (318.25, 333.00)	0.339
MCH(pg), Median (IQR)	29.90 (28.80, 31.20)	29.90 (28.80, 31.20)	30.60 (29.02, 31.60)	0.177
PLT count(10^9^/L), Median (IQR)	203.00 (149.00, 275.00)	205.00 (151.00, 276.00)	171.00 (106.00, 238.00)	**0.006**
PDW(fL), Median (IQR)	11.90 (10.20, 15.40)	12.00 (10.20, 15.40)	11.60 (10.22, 15.28)	0.618
MPV(fL), Median (IQR)	10.10 (9.30, 11.00)	10.10 (9.20, 11.00)	10.15 (9.53, 11.17)	0.499
Neutrophils percentage(%), Median (IQR)	80.20 (70.65, 88.00)	79.90 (70.20, 87.80)	85.55 (75.40, 91.77)	**0.003**
Neutrophils count(10^9^/L), Median (IQR)	6.94 (4.58, 10.61)	6.88 (4.58, 10.43)	7.61 (4.72, 12.44)	0.315
Monocytes percentage (%), Median (IQR)	6.00 (4.40, 7.80)	6.00 (4.50, 7.80)	5.20 (2.55, 7.30)	**0.026**
Monocytes count(10^9^/L), Median (IQR)	0.51 (0.37, 0.73)	0.52 (0.38, 0.73)	0.42 (0.22, 0.71)	**0.026**
Lymphocytes percentage(%), Median (IQR)	11.40 (6.30, 19.40)	11.60 (6.50, 19.80)	7.80 (4.23, 14.62)	**0.004**
Lymphocytes count(10^9^/L), Median (IQR)	1.02 (0.66, 1.52)	1.04 (0.67, 1.52)	0.86 (0.49, 1.24)	**0.007**
Basophils percentage (%), Median (IQR)	0.20 (0.10, 0.30)	0.20 (0.10, 0.30)	0.20 (0.10, 0.30)	0.077
Basophils count (10^9^/L), Median (IQR)	0.02 (0.01, 0.03)	0.02 (0.01, 0.03)	0.01 (0.00, 0.04)	0.111
Eosinophils percentage (%), Median (IQR)	0.50 (0.10, 1.40)	0.50 (0.10, 1.50)	0.20 (0.00, 0.50)	**0.003**
Eosinophils count (10^9^/L), Median (IQR)	0.06 (0.01, 0.14)	0.06 (0.01, 0.15)	0.01 (0.00, 0.06)	**< 0.001**
NLR, Median (IQR)	6.95 (3.66, 13.76)	6.78 (3.56, 13.33)	10.14 (5.07, 20.96)	**0.010**
PLR, Median (IQR)	71.91 (47.86, 115.9)	71.47 (47.70, 114.58)	83.70 (52.81, 120.48)	0.115
PCT, Median (IQR)	0.13 (0.06, 0.62)	0.13 (0.06, 0.55)	0.45 (0.07, 3.07)	**0.001**
Urea (mmol/L), Median (IQR)	5.95 (4.44, 8.52)	5.95 (4.38, 8.21)	7.64 (5.52, 16.91)	**< 0.001**
Creatinine (umol/L), Median (IQR)	55.25 (44.20, 77.25)	55.25 (44.20, 75.70)	72.30 (46.65, 140.02)	**0.011**
UA (umol/L), Median (IQR)	211.00 (155.00, 290.00)	211.00 (155.00, 285.00)	234.50 (153.75, 393.00)	0.092
CYSC (mg/L), Median (IQR)	1.04 (0.85, 1.36)	1.04 (0.85, 1.33)	1.11 (0.88, 2.04)	0.128
Serum CO_2_ (mmol/L), Median (IQR)	25.20 (24.40, 26.00)	25.30 (24.60, 26.00)	25.00 (22.00, 25.30)	0.092
TBIL (umol/L), Median (IQR)	9.20 (6.30, 13.80)	9.20 (6.10, 13.20)	13.60 (9.20, 21.60)	**< 0.001**
DBIL (umol/L), Median (IQR)	4.50 (3.10, 6.50)	4.50 (3.10, 6.30)	6.40 (4.60, 11.33)	**< 0.001**
AST (U/L), Median (IQR)	22.40 (16.40, 33.30)	22.40 (16.40, 32.40)	24.40 (16.00, 44.82)	0.275
ALT (U/L), Median (IQR)	19.40 (12.60, 32.25)	19.40 (12.30, 31.20)	22.50 (13.62, 41.90)	0.140
TBA (U/L), Median (IQR)	3.30 (2.10, 5.85)	3.30 (2.10, 5.90)	2.85 (1.80, 5.62)	0.360
TP (g/L), Mean (SD)	60.82 ± 7.92	61.11 ± 7.73	56.92 ± 9.35	**0.001**
ALB (g/L), Mean (SD)	35.00 (31.30, 38.60)	35.20 (31.40, 38.60)	33.30 (29.28, 37.15)	0.088
AFU (U/L), Median (IQR)	18.30 (15.90, 21.25)	18.20 (15.90, 21.30)	18.40 (16.48, 21.17)	0.366
CHE (U/L), Median (IQR)	4811.50 (3645.00, 6204.00)	4811.50 (3726.00, 6236.00)	4156.00 (2853.75, 5644.25)	**0.012**
PA (mg/dL), Median (IQR)	153.00 (118.35, 198.00)	153.00 (119.90, 198.60)	150.00 (97.60, 183.30)	0.151
α-HBDH (U/L), Median (IQR)	161.00 (148.50, 176.00)	161.00 (148.00, 174.00)	163.00 (161.00, 185.50)	0.170
LDH (U/L), Median (IQR)	205.00 (195.50, 226.50)	205.00 (193.00, 226.00)	208.00 (205.00, 233.50)	0.298
ALP (U/L), Median (IQR)	85.00 (66.00, 106.00)	85.00 (67.00, 106.00)	76.50 (58.00, 106.00)	0.062
γ-GGT (U/L), Median (IQR)	28.90 (16.80, 55.00)	29.00 (17.00, 55.00)	28.50 (16.50, 45.50)	0.625
ADA (U/L), Median (IQR)	12.00 (10.00, 13.90)	12.00 (10.00, 13.90)	11.80 (8.75, 13.73)	0.746
SO_2_ (%), Median (IQR)	97.50 (97.20, 97.60)	97.50 (97.00, 97.70)	97.60 (97.30, 97.90)	0.826
pH, Median (IQR)	7.42 (7.41, 7.42)	7.42 (7.41, 7.42)	7.40 (7.40, 7.41)	0.599
HCO_3_^-^(mmol/L), Median (IQR)	27.40 (27.20, 28.40)	27.40 (27.20, 28.30)	27.50 (27.40, 28.60)	0.196
BE (mmol/L), Median (IQR)	3.15 (2.95, 3.93)	3.15 (2.90, 3.90)	3.18 (3.15, 4.03)	0.286
PO_2_ (mmHg), Median (IQR)	90.60 (87.95, 93.25)	90.60 (87.20, 93.80)	90.70 (90.60, 90.80)	0.540
PCO_2_ (mmHg), Median (IQR)	41.80 (41.80, 43.00)	41.80 (41.70, 43.20)	42.00 (41.80, 42.30)	0.218
AG (mmHg), Median (IQR)	10.70 (10.30, 11.05)	10.70 (10.20, 11.10)	10.70 (10.70, 10.70)	0.483
Lac (mmol/L), Median (IQR)	1.70 (1.60, 1.72)	1.68 (1.60, 1.70)	1.71 (1.70, 1.73)	0.051
TG (mmol/L), Median (IQR)	1.09 (0.83, 1.50)	1.09 (0.82, 1.48)	1.17 (0.87, 1.69)	0.188
CHOL (mmol/L), Median (IQR)	3.41 (2.76, 4.06)	3.41 (2.80, 4.06)	2.84 (2.03, 3.93)	**0.001**
LDL (mmol/L), Median (IQR)	1.80 (1.80, 1.80)	1.80 (1.80, 1.80)	1.78 (1.03, 1.80)	**0.018**
HDL (mmol/L), Median (IQR)	0.94 (0.94, 0.94)	0.94 (0.94, 0.94)	0.92 (0.70, 0.94)	**0.007**
TT (s), Median (IQR)	17.00 (16.00, 18.20)	17.00 (16.00, 18.20)	17.05 (15.80, 18.08)	0.833
PT (s), Median (IQR)	13.40 (12.40, 14.70)	13.40 (12.40, 14.60)	14.60 (12.83, 16.08)	**0.002**
INR, Median (IQR)	1.10 (1.03, 1.19)	1.10 (1.03, 1.18)	1.18 (1.09, 1.29)	**< 0.001**
APTT (s), Median (IQR)	32.90 (29.10, 38.35)	32.90 (29.10, 38.20)	33.60 (30.70, 40.35)	0.191
D-dimer (mg/L), Median (IQR)	1.85 (0.88, 3.71)	1.85 (0.86, 3.50)	2.60 (1.39, 6.76)	**0.002**
Fibrinogen (g/L), Median (IQR)	4.19 (3.20, 5.20)	4.19 (3.26, 5.16)	4.00 (2.61, 5.52)	0.249
Phosphate (mmol/L), Median (IQR)	1.04 (0.85, 1.22)	1.04 (0.86, 1.23)	0.92 (0.74, 1.15)	**0.030**
Sodium (mmol/L), Median (IQR)	139.30 (136.70, 142.10)	139.30 (136.60, 141.90)	140.45 (137.32, 145.83)	**0.019**
Magnesium (mmol/L), Median (IQR)	0.88 (0.81, 0.95)	0.88 (0.81, 0.94)	0.90 (0.78, 0.99)	0.670
Potassium (mmol/L), Median (IQR)	4.05 (3.77, 4.34)	4.05 (3.78, 4.34)	4.00 (3.69, 4.37)	0.414
Calcium (mmol/L), Median (IQR)	2.17 (2.08, 2.26)	2.17 (2.09, 2.26)	2.09 (1.98, 2.19)	**< 0.001**
CK (U/L), Median (IQR)	50.00 (29.50, 97.50)	50.00 (29.00, 91.00)	79.00 (31.75, 275.25)	**0.010**
Treatment, n (%)				
Endotracheal intubation, *n* (%)				**< 0.001**
No	611 (73.88)	585 (76.07)	26 (44.83)	
Yes	216 (26.12)	184 (23.93)	32 (55.17)	
Mechanical ventilation, *n* (%)				**< 0.001**
No	510 (61.67)	489 (63.59)	21 (36.21)	
Yes	317 (38.33)	280 (36.41)	37 (63.79)	
Ventilator weaning failure, *n* (%)				**0.008**
No	808 (97.70)	755 (98.18)	53 (91.38)	
Yes	19 (2.30)	14 (1.82)	5 (8.62)	
Vasoactive medications, *n* (%)				**< 0.001**
No	757 (91.54)	714 (92.85)	43 (74.14)	
Yes	70 (8.46)	55 (7.15)	15 (25.86)	
CRRT use, *n* (%)				**< 0.001**
No	775 (93.71)	732 (95.19)	43 (74.14)	
Yes	52 (6.29)	37 (4.81)	15 (25.86)	
ECMO use, *n* (%)				1.000
No	824 (99.64)	766 (99.61)	58 (100.00)	
Yes	3 (0.36)	3 (0.39)	0 (0.00)	
Anticoagulant usage, *n* (%)				0.234
No	608 (73.52)	561 (72.95)	47 (81.03)	
Yes	219 (26.48)	208 (27.05)	11 (18.97)	
Prior antibiotic exposure, *n* (%)				**0.032**
No	782 (73.52)	731 (95.06)	51 (87.93)	
Yes	45 (26.48)	38 (4.94)	7 (12.07)	
Immunosuppression, *n* (%)				1.000
No	852 (99.76)	767 (99.76)	58 (100.00)	
Yes	2 (0.24)	2 (0.26)	0 (0.00)	
Invasive catheters, *n* (%)				**0.028**
No	783 (94.68)	732 (95.19)	51 (87.93)	
Yes	44 (5.32)	37 (4.81)	7 (12.07)	
Comorbidity, *n* (%)				
Hyperlipidemia, *n* (%)				0.614
No	814 (98.43)	757 (98.44)	57 (98.28)	
Yes	13 (1.57)	12 (1.56)	1 (1.72)	
Hyperlactatemia, *n* (%)				0.442
No	819 (99.03)	762 (99.09)	57 (98.28)	
Yes	8 (0.97)	7 (0.91)	1 (1.72)	
Hypertension, *n* (%)				0.672
No	400 (48.37)	374 (48.63)	26 (44.83)	
Yes	427 (51.63)	395 (51.37)	32 (55.17)	
Diabetes mellitus, *n* (%)				0.239
No	630 (76.18)	590 (76.72)	40 (68.97)	
Yes	197 (23.82)	179 (23.28)	18 (31.03)	
Diabetes-related complications, *n* (%)				1.000
No	801 (96.86)	744 (96.75)	57 (98.28)	
Yes	26 (3.14)	25 (3.25)	1 (1.72)	
Coronary heart disease, *n* (%)				0.685
No	705 (85.25)	654 (85.05)	51 (87.93)	
Yes	122 (14.75)	115 (14.95)	7 (12.07)	
Atrial fibrillation, *n* (%)				0.293
No	768 (92.87)	716 (93.11)	52 (89.66)	
Yes	59 (7.13)	53 (6.89)	6 (10.34)	
History of CKD, *n* (%)				**0.005**
No	803 (97.10)	751 (97.66)	52 (89.66)	
Yes	24 (2.90)	18 (2.34)	6 (10.34)	
Heart failure, *n* (%)				0.306
No	751 (90.81)	701 (91.16)	50 (86.21)	
Yes	76 (9.19)	68 (8.84)	8 (13.79)	
Anemia, *n* (%)				0.355
No	646 (78.11)	604 (78.54)	42 (72.41)	
Yes	181 (21.89)	165 (21.46)	16 (27.59)	
History of COPD, *n* (%)				**< 0.001**
No	603 (72.91)	576 (74.90)	27 (46.55)	
Yes	224 (27.09)	193 (25.10)	31 (53.45)	
Infection site, n (%)				
Liver abscess, *n* (%)				1.000
No	826 (99.88)	768 (99.87)	58 (100.00)	
Yes	1 (0.12)	1 (0.13)	0 (0.00)	
Cholecystitis, *n* (%)				0.364
No	809 (97.82)	753 (97.92)	56 (96.55)	
Yes	18 (2.18)	16 (2.08)	2 (3.45)	
Cholangitis, *n* (%)				1.000
No	822 (99.40)	764 (99.35)	58 (100.00)	
Yes	5 (0.60)	5 (0.65)	0 (0.00)	
Intra-abdominal infection, *n* (%)				**0.020**
No	818 (98.91)	763 (99.22)	55 (94.83)	
Yes	9 (1.09)	6 (0.78)	3 (5.17)	
Pneumonia, *n* (%)				0.670
No	212 (25.63)	199 (25.88)	13 (22.41)	
Yes	615 (74.37)	570 (74.12)	45 (77.59)	
Urinary tract infection, *n* (%)				**0.030**
No	792 (95.77)	740 (96.23)	52 (89.66)	
Yes	35 (4.23)	29 (3.77)	6 (10.34)	
Intracranial infection, *n* (%)				1.000
No	793 (95.89)	737 (95.84)	56 (96.55)	
Yes	34 (4.11)	32 (4.16)	2 (3.45)	
Acute organ injury, n (%)				
Acute liver injury, *n* (%)				0.138
No	716 (86.58)	670 (87.13)	46 (79.31)	
Yes	111 (13.42)	99 (12.87)	12 (20.69)	
Acute kidney injury, *n* (%)				0.052
No	797 (96.37)	744 (96.75)	53 (91.38)	
Yes	30 (3.63)	25 (3.25)	5 (8.62)	
Skull and brain injury, *n* (%)				0.853
No	728 (88.03)	676 (87.91)	52 (89.66)	
Yes	99 (11.97)	93 (12.09)	6 (10.34)	
MODS, *n* (%)				**0.006**
No	821 (99.27)	766 (99.61)	55 (94.83)	
Yes	6 (0.73)	3 (0.39)	3 (5.17)	
Stroke, *n* (%)				0.937
No	588 (71.10)	546 (71.00)	42 (72.41)	
Yes	239 (28.90)	223 (29.00)	16 (27.59)	
Cardiac arrest, *n* (%)				0.257
No	813 (98.31)	757 (98.44)	56 (96.55)	
Yes	14 (1.69)	12 (1.56)	2 (3.45)	
Altered mental status, *n* (%)				0.438
No	706 (85.37)	659 (85.70)	47 (81.03)	
Yes	121 (14.63)	110 (14.30)	11 (18.97)	
Metabolic encephalopathy, *n* (%)				1.000
No	824 (99.64)	766 (99.61)	58 (100.00)	
Yes	3 (0.36)	3 (0.39)	0 (0.00)	
Hepatic encephalopathy, *n* (%)				1.000
No	826 (99.88)	768 (99.87)	58 (100.00)	
Yes	1 (0.12)	1 (0.13)	0 (0.00)	
Ventricular fibrillation, *n* (%)				0.196
No	824 (99.64)	767 (99.74)	57 (98.28)	
Yes	3 (0.36)	2 (0.26)	1 (1.72)	
Complication, n (%)				
Coagulopathy, *n* (%)				**0.005**
No	787 (95.16)	737 (95.84)	50 (86.21)	
Yes	40 (4.84)	32 (4.16)	8 (13.79)	
DIC, *n* (%)				0.253
No	823 (99.52)	766 (99.61)	57 (98.28)	
Yes	4 (0.48)	3 (0.39)	1 (1.72)	
ARDS, *n* (%)				**< 0.001**
No	477 (57.68)	463 (60.21)	14 (24.14)	
Yes	350 (42.32)	306 (39.79)	44 (75.86)	

Bold values indicate statistical significance (P < 0.05).

### 3.3 Selection of predictor variables

**Figure 2:**
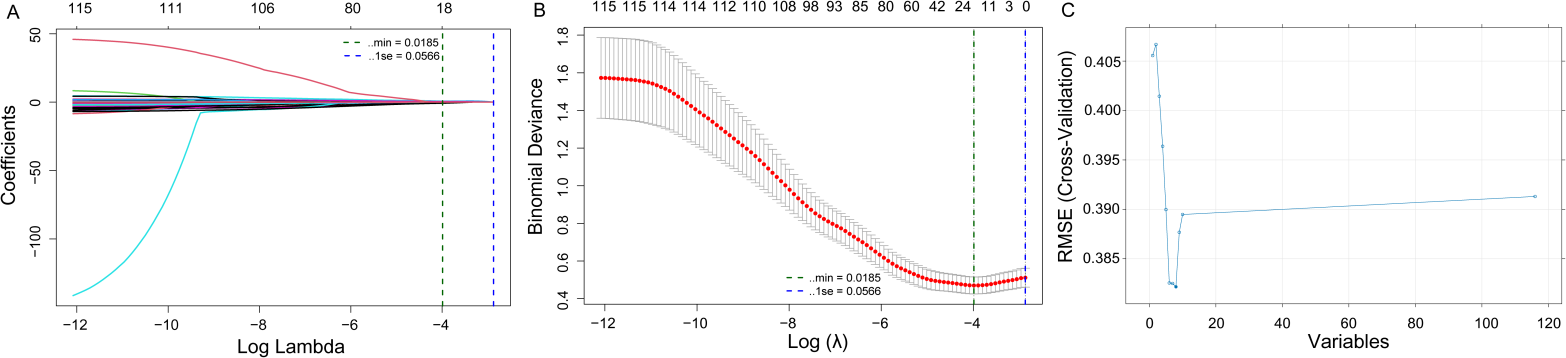
Feature selection process for variables included in the prediction model. **(A)** LASSO regression path showing the coefficients of variables across different values of the regularization parameter (λ). **(B)** Cross-validation error plot for selecting the optimal λ in LASSO. The vertical dashed line represents the optimal λ where the minimal cross-validation error is achieved. **(C)** Variable importance based on the SVM-RFE algorithm. LASSO, least absolute shrinkage and selection operator; RMSE, root mean square error; SE, standard error; CV, cross-validation.

Feature selection integrated LASSO regression and SVM-RFE algorithms. LASSO regression identified 18 predictive variables, including laboratory parameters (urea, uric acid, TBIL, creatinine, calcium, RDW-SD, NLR), coagulation profiles (PT), and clinical variables encompassing organ-dysfunction markers (MODS, ARDS, coagulopathy, intra-abdominal infection) and therapeutic interventions (invasive catheters, endotracheal intubation, vasoactive medications, prior antibiotic exposure, COPD) (**Figures 2A,B**). SVM-RFE identified 8 key predictors, including invasive catheters, prior antibiotic exposure, COPD, intra-abdominal infection, RDW-SD, WBC, UA, and calcium (**Figure 2C**). Cross-validation analysis identified 6 consistent predictive factors: invasive catheters, prior antibiotic exposure, COPD, intra-abdominal infection, RDW-SD, and calcium, all predictors above exhibited acceptable multicollinearity (all VIFs < 5) (**Supplementary Table 4**), which were incorporated into the final model.

### 3.4 Model development and performance comparison

**Figure 3:**
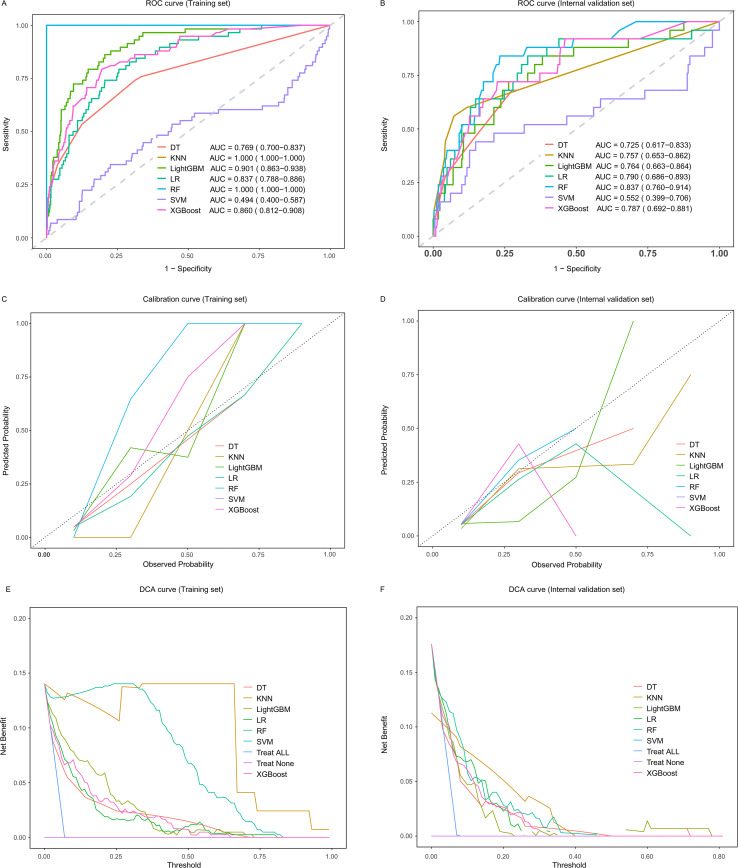
Algorithm performance comparison for sepsis prediction modeling in patients with MDR-PA infections across training (N = 827) and internal validation datasets (N = 355). ROC curve evaluation **(A, B)**, calibration curve analysis **(C, D)**, and DCA assessment **(E, F)** demonstrate seven ML algorithm effectiveness in training (N = 827) and internal validation datasets (N = 355). ROC, receiver operating characteristic; AUC, area under the curve; DCA, decision curve analysis; DT, decision tree; KNN, k-nearest neighbor; LightGBM, light gradient boosting machine; LR, logistic regression; RF, random forest; SVM, support vector machine; XGBoost, extreme gradient boosting.

Seven machine learning methodologies were developed using ten rounds of 10-fold internal cross-validation procedures to determine the optimal hyperparameters for each algorithm (**Supplementary Table 5**). Among the training dataset, random forest modeling achieved optimal discrimination and calibration characteristics, yielding an area under the curve of 1.000 and Brier score of 0.019. Random forest demonstrated the highest performance across multiple evaluation metrics: accuracy (1.000), sensitivity (1.000), specificity (1.000), PPV (1.000), NPV (1.000), precision (1.000), recall (1.000), and F1 score (1.000) (**Figures 3A,C**; **Table 3**). The DCA showed that if the threshold probability for sepsis is between 0% and 85%, using the random forest model adds more net benefit than treating all or no patients (**Figure 3E**).

**Table 3:** Predictive performance comparison of the seven types of machine learning algorithms in the training and internal validation cohorts.

Model	Train cohort									
	AUC	Accuracy	Sensitivity	Specificity	PPV	NPV	Precision	Recall	F1 score	Brier score
Logistic Regression	0.830	0.682	0.845	0.670	0.162	0.983	0.162	0.845	0.271	0.055
Decision Tree	0.769	0.937	0.207	0.992	0.667	0.943	0.667	0.207	0.316	0.055
Random Forest	1.000	1.000	1.000	1.000	1.000	1.000	1.000	1.000	1.000	0.019
XGBoost	0.860	0.804	0.640	0.797	0.193	0.967	0.193	0.640	0.296	0.053
SVM	0.494	0.255	0.655	0.225	0.060	0.896	0.060	0.655	0.110	0.065
KNN	1.000	1.000	1.000	1.000	1.000	1.000	1.000	1.000	1.000	0.010
LightGBM	0.901	0.799	0.862	0.795	0.240	0.987	0.240	0.862	0.376	0.050
Test Cohort										
Logistic Regression	0.796	0.699	0.760	0.694	0.158	0.974	0.158	0.760	0.262	0.062
Decision Tree	0.725	0.930	0.500	0.994	0.500	0.934	0.500	0.500	0.138	0.060
Random Forest	0.837	0.930	0.800	0.985	0.500	0.942	0.500	0.800	0.286	0.057
XGBoost	0.787	0.786	0.640	0.797	0.193	0.967	0.193	0.640	0.296	0.060
SVM	0.552	0.239	0.680	0.206	0.061	0.895	0.061	0.680	0.112	0.066
KNN	0.769	0.918	0.320	0.964	0.400	0.949	0.400	0.320	0.356	0.062
LightGBM	0.764	0.775	0.600	0.788	0.176	0.963	0.176	0.600	0.273	0.062

AUC, area under the receiver operating characteristic curve; PPV, positive predictive value; NPV, negative predictive value; XGBoost, extreme gradient boosting; SVM, support vector machine; KNN, k-nearest neighbors; LightGBM, light gradient boosting machine.

**Figure 4:**
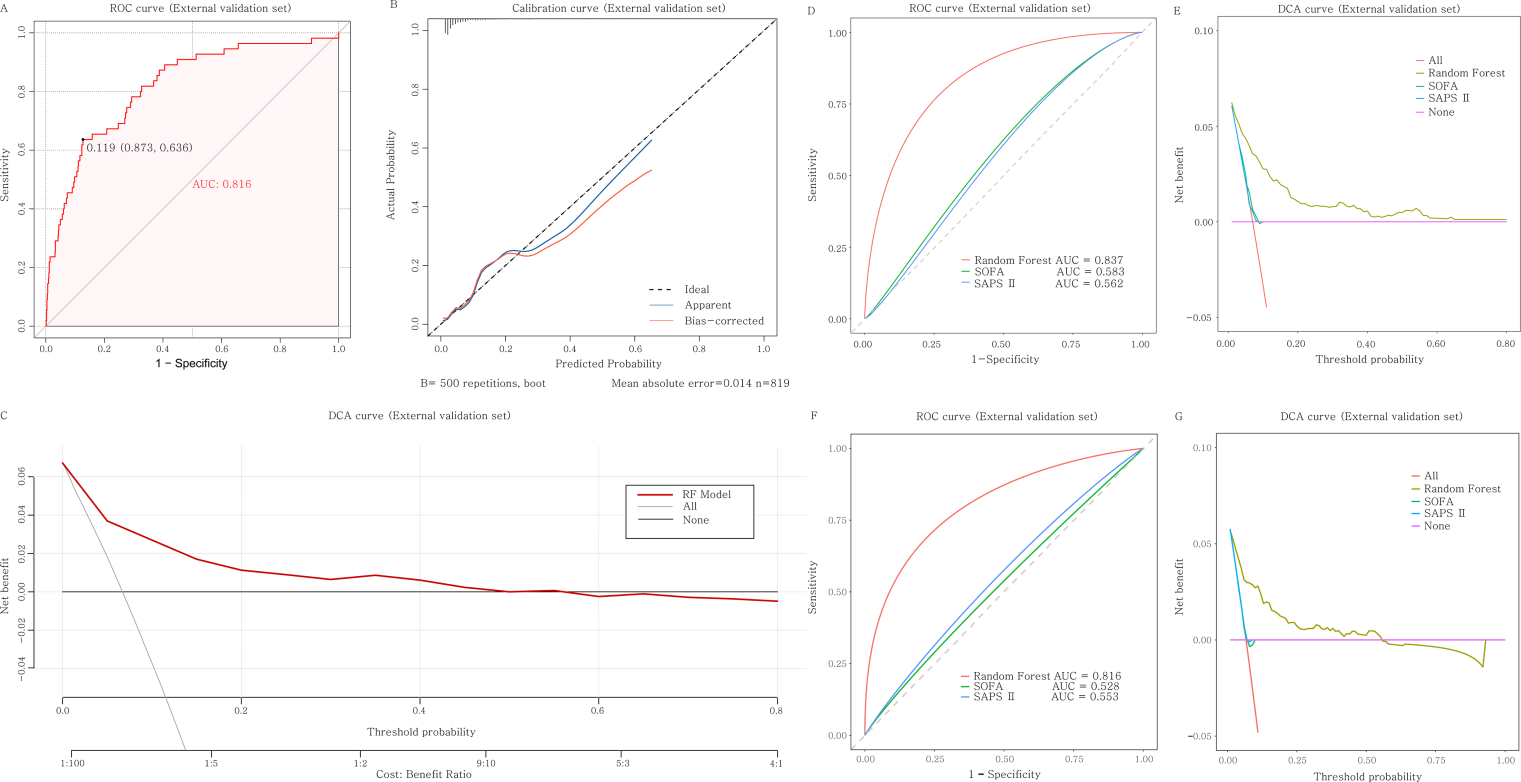
Performance evaluation and comparison of the random forest model. **(A)** ROC curve of the RF model in the external validation set (N = 819). **(B)** Calibration curve of the RF model in the external validation set (N = 819). **(C)** DCA curve of the RF model in the external validation set (N = 819). **(D)** ROC curves comparing the RF model, SOFA, and SAPS II scores in the internal validation set (N = 355). **(E)** DCA curves comparing the RF model, SOFA, and SAPS II scores in the internal validation set (N = 355). **(F)** ROC curves comparing the RF model, SOFA, and SAPS II scores in the external validation set (N = 819). **(G)** DCA curves comparing the RF model, SOFA, and SAPS II scores in the external validation set (N = 819). The y-axis measures the net benefit. The red line represents the RF model, the gray line represents the assumption that all patients have the outcome (“All”), and the black line represents the assumption that no patients have the outcome (“None”). ROC, receiver operating characteristic; AUC, area under the curve; DCA, decision curve analysis; SOFA, sequential organ failure assessment score; SAPS II, simplified acute physiology score II; RF, random forest; CI, confidence interval.

During internal validation testing, the random forest methodology exhibited the strongest predictive performance, generating an AUC of 0.837 alongside a Brier score of 0.057. The random forest model produced notable evaluation metrics: accuracy (0.930), sensitivity (0.800), specificity (0.985), positive predictive value (0.500), negative predictive value (0.942), precision (0.500), recall (0.800), and F1-score (0.286) (**Figures 3B,D**, **Table 3**). External validation assessment utilized an independent dataset comprising 819 cases from the Affiliated Huai’an Hospital of Xuzhou Medical University. Despite inter-institutional patient characteristic disparities (**Table 1**), random forest modeling preserved excellent predictive capacity, achieving an AUC of 0.816 (**Figure 4A**). The calibration plot exhibited appropriate fit quality (Hosmer-Lemeshow statistic, P = 0.896) (**Figure 4B**). Initial clinical decision curve analysis confirmed that if the threshold probability is between 0% and 50%, using this model adds more net benefit than treating all or no patients (**Figure 4C**).

Further comparative analysis was conducted to evaluate our model’s predictive performance against existing scoring systems. In the internal validation set, the RF model significantly outperformed both SOFA (AUC = 0.583, 95% CI: 0.506–0.655) and SAPS II scores (AUC = 0.562, 95% CI: 0.492–0.630) in sepsis risk prediction (both P < 0.05, **Figure 4D**) and showed greater clinical utility when the probability threshold exceeded 0.05 (**Figure 4E**). To further validate its clinical robustness, we extended this comparison to the external cohort. Consistent with internal findings, the RF model maintained its significant superiority over both SOFA (AUC = 0.528, 95% CI: 0.446–0.611) and SAPS II (AUC = 0.553, 95% CI: 0.473–0.632) (**Figure 4F**), and demonstrated a substantially higher net clinical benefit across a wide range of threshold probabilities (**Figure 4G**). This comprehensive superiority in both discrimination and clinical utility supports the potential value of our RF model in guiding clinical decision-making for MDR-PA infection patients at risk of sepsis.

### 3.5 Model explanation

**Figure 5:**
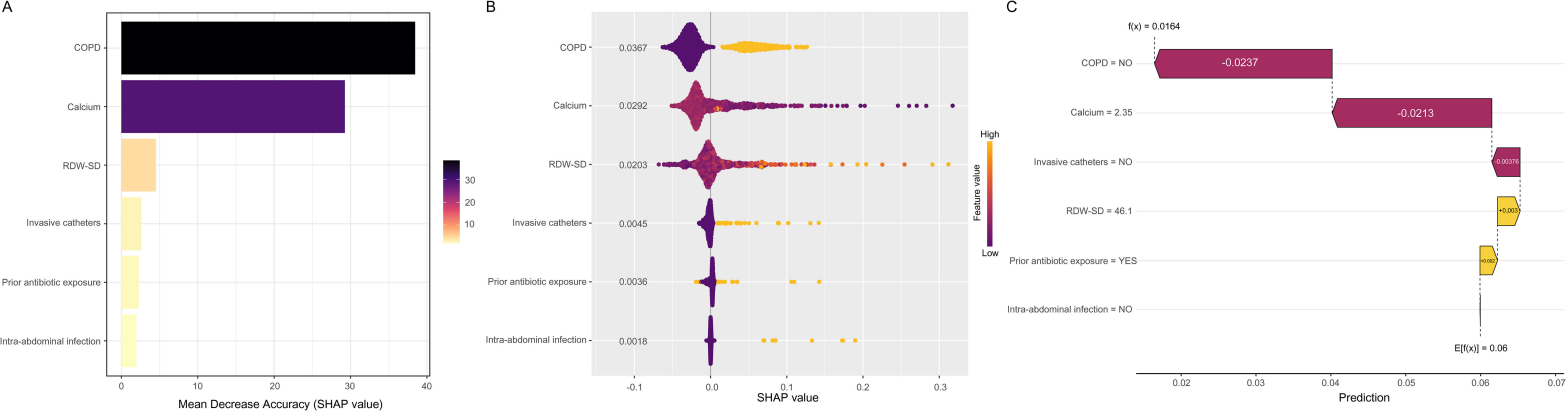
SHAP-based model explanation for global and individual-level interpretability. **(A)** SHAP importance summary. Feature contributions are ranked by mean SHAP magnitude in descending sequence. **(B)** SHAP distribution plot. Higher SHAP values correspond to increased sepsis probability. Each point represents patient-specific SHAP values per feature, with orange indicating higher feature magnitudes and purple showing lower magnitudes. Point stacking demonstrates data distribution density. **(C)** SHAP contribution waterfall. Individual feature impacts on prediction for patient five using random forest methodology. Orange bars denote positive predictive effects, purple bars represent negative impacts. Feature magnitudes are paired with SHAP coefficients, highlighting significant variables: COPD (- 0.0237), calcium levels (- 0.0213), invasive catheters (- 0.00376), RDW-SD (+ 0.003), prior antibiotic exposure (+ 0.002), and intra-abdominal infection (- 0.001). SHAP, SHapley Additive exPlanations; RDW-SD, red blood cell distribution width-standard deviation; COPD, chronic obstructive pulmonary disease.

Since healthcare practitioners typically resist adoption of non-interpretable predictive models, SHAP methodology was utilized to enhance algorithmic transparency by calculating individual feature contributions to predictive outputs. This explainability approach encompasses two analytical modalities: comprehensive model-wide feature interpretation and detailed individual-patient explanation. Comprehensive model explanation reveals the complete algorithmic framework. SHAP variable ranking (**Figure 5A**) evaluated feature importance through mean SHAP score calculation displayed in decreasing order: COPD, calcium measurements, RDW-SD indices, invasive catheters, prior antibiotic exposure, and intra-abdominal infections represented the six principal predictive components. The SHAP distribution chart (**Figure 5B**) visually depicts directional effects and strength of each factor on model predictions: invasive catheters, calcium deficiency, COPD formation, prior antibiotic exposure, elevated RDW-SD levels, and intra-abdominal infections notably increased sepsis likelihood. Moreover, SHAP dependency plots facilitate comprehension of individual feature influences on predictive model outputs.

Local interpretation facilitates understanding of individual prediction mechanisms through calculation and visualization of feature contributions for specific samples. The SHAP waterfall diagram (**Figure 5C**) illustrates feature-level contributions to sepsis prediction for six patients with MDR-PA infection. Individual feature values and corresponding SHAP coefficients demonstrate positive or negative predictive influences. RDW-SD (46.1) and prior antibiotic exposure (YES) contributed substantially positive values of + 0.003 and + 0.002, respectively. Conversely, absence of COPD, calcium concentration (2.35), no invasive catheters and no intra-abdominal infection presence yielded significant negative contributions of - 0.0237, - 0.0213, - 0.0037 and -0.001, respectively. Through cumulative SHAP value aggregation, the waterfall visualization demonstrates prediction formation processes for individual patients, providing comprehensive insight into algorithmic decision-making pathways.

### 3.6 Implementation of the web calculator

**Figure 6:**
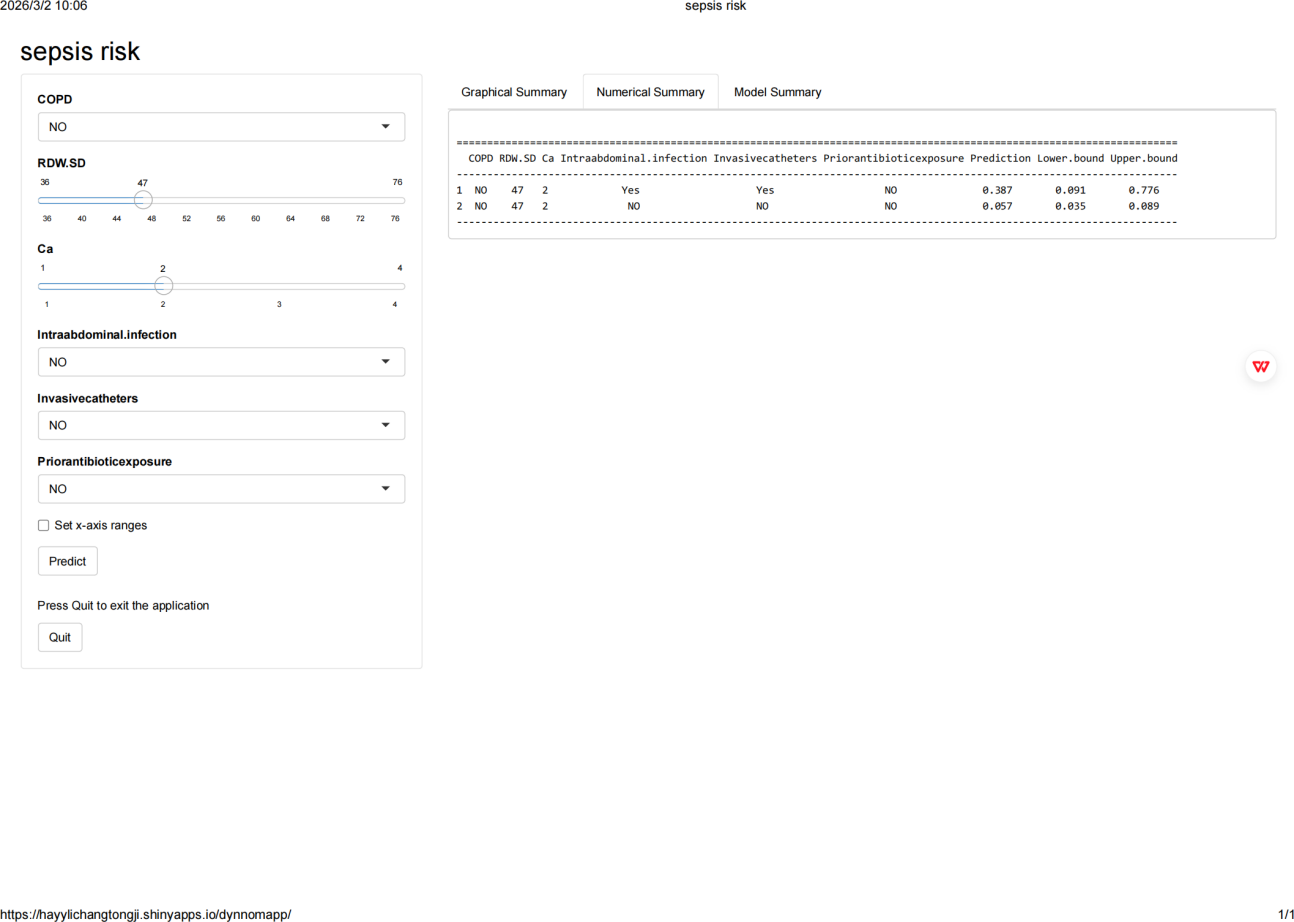
Screenshot of the web-based dynamic nomogram for predicting the risk of sepsis. The online calculator allows users to input specific clinical variables (invasive catheters, prior antibiotic exposure, calcium, COPD, RDW-SD, and intra-abdominal infection) on the left panel. The right panel displays the predicted probability of sepsis risk along with the 95% confidence interval (Lower bound and Upper bound) in both graphical and numerical formats. The tool is accessible at https://hayylichangtongji.shinyapps.io/dynnomapp/. Ca, calcium; COPD, chronic obstructive pulmonary disease; RDW-SD, red blood cell distribution width-standard deviation; CI, confidence interval.

As depicted in **Figure 6**, the developed prediction algorithm was incorporated into a user-friendly web interface to facilitate clinical utilization. By inputting the actual values of the 6 features required for the model, the application can automatically predict the risk of sepsis in patients with MDR-PA infection. The web application can be accessed online at the following link: https://hayylichangtongji.shinyapps.io/dynnomapp/.

### 3.7 Sensitivity analysis of model performance

In the internal test cohort, the AUC of the model using MICE was 0.837 (95% CI: 0.760–0.914), while the complete case analysis with an AUC of 0.806 (95% CI: 0.704–0.908) (**Supplementary Table 6** and **Supplementary Figure 2**). In the external validation cohort, the AUC values were 0.816 (95% CI: 0.746–0.891) for the MICE and 0.804 (95% CI: 0.738–0.871) for the complete case analysis (**Supplementary Table 7** and **Supplementary Figure 3**). These results indicate that the two methods achieved relatively similar AUC values and maintained high model performance overall.

## 4 Discussion

This study represents the first comprehensive multicenter investigation developing an interpretable machine learning model for predicting sepsis in patients with multidrug-resistant *Pseudomonas aeruginosa* (MDR-PA) infections. By analyzing 2,001 patients, we developed a robust random forest model that achieved excellent discrimination (AUC = 0.837) in internal validation test using only six routine clinical variables. The sepsis incidence of ~7% across both derivation and validation cohorts underscores the significant burden of MDR-PA (Vena et al., 2020). Unlike general sepsis models (Fleuren et al., 2020), our pathogen-specific approach targets the unique risks associated with this resistant organism, providing a stable and generalizable tool for early risk stratification.

The selection of COPD, invasive catheters, prior antibiotic exposure, and intra-abdominal infection as key predictors highlights the interplay between host vulnerability and iatrogenic risks (Rudin, 2019; Thelagathoti et al., 2025). COPD represent states of chronic immunosuppression and structural compromise; the latter specifically acts as a reservoir for *P. aeruginosa* colonization and biofilm formation (Zoccali and Mallamaci, 2023; Opron et al., 2024). Invasive catheters (e.g., central venous lines, urinary catheters) breach natural anatomical barriers, providing direct entry points for MDR-PA to invade the bloodstream and trigger sepsis (Pitiriga et al., 2024). Similarly, prior antibiotic exposure disrupts the gut microbiota, promoting the dominance of resistant pathogens like MDR-PA and increasing host susceptibility to severe infection (Grießhammer et al., 2025). Similarly, intra-abdominal infections are distinct drivers of sepsis due to anatomical complexity and difficulties in achieving adequate antibiotic penetration (Martin-Loeches et al., 2019; Bova et al., 2024). Together, these factors delineate a specific phenotype of patients with diminished physiological reserve who are prone to rapid deterioration upon MDR-PA infection.

Our model uniquely integrates hypocalcemia and RDW-SD as early warning signals of systemic dysregulation. Hypocalcemia likely reflects underlying malnutrition or the initial phase of the systemic inflammatory response (Minasi et al., 2023; Fernandes and Pereira, 2024). Specifically, the severe systemic inflammatory response and potential cytokine storm triggered by MDR-PA infection can suppress parathyroid hormone secretion or induce the intracellular shift and precipitation of calcium ions. Consequently, hypocalcemia manifests as a critical metabolic early warning signal even before overt organ failure occurs. Concurrently, elevated RDW-SD serves as a robust marker of chronic inflammation and oxidative stress (Yan et al., 2023). By capturing these subtle metabolic and hematological alterations, the model can detect “pre-sepsis” states that precede the gross organ dysfunction typically required to trigger traditional alarms.

A critical finding is our model’s superiority over both traditional scoring systems and general-purpose machine learning algorithms. In our internal validation, SOFA and SAPS II performed poorly (AUC ~0.56), We acknowledge that SOFA and SAPS II are inherently lagging indicators designed to assess established organ dysfunction rather than to predict its future onset. Our comparison is not intended to claim algorithmic superiority in the same clinical domain, but rather to highlight the critical lack of “early prediction” tools in current practice. Our RF model can identify high-risk patients before the SOFA score significantly elevates (i.e., before clinical diagnosis), demonstrating the precise clinical value of ML-based early warning systems (Singer et al., 2016). Similarly, while general sepsis models, such as those based on the MIMIC-III database or recent advanced frameworks like MedAI (Shan et al., 2024), report high accuracy, they often depend on high-frequency ICU monitoring data or hundreds of variables, limiting their utility in general wards (Desautels et al., 2016; Moor et al., 2021). By focusing specifically on the MDR-PA cohort, our model achieves high precision with minimal data requirements, effectively bridging the gap between generic screening tools and the specific needs of managing resistant infections.

The choice of the random forest algorithm allowed for the handling of complex, non-linear relationships among clinical variables (Silva et al., 2025), which we further elucidated using SHAP analysis. This interpretability addresses the “black box” limitation of artificial intelligence in healthcare, enabling clinicians to visualize individual risk contributors, enabling clinicians to visualize individual risk contributors (Ghassemi et al., 2021; Cui et al., 2025). To facilitate immediate clinical translation, we deployed a web-based calculator. This tool allows for real-time risk assessment without specialized software, supporting timely decision-making regarding surveillance intensity and early intervention (Sendak et al., 2020a, b).

Our study has several limitations. First, the retrospective observational design inherently introduces potential selection and information biases. Although we utilized an independent multicenter cohort for external validation, unmeasured confounders may still exist. Furthermore, our cohort was exclusively comprised of Chinese patients from the same geographic region, which inherently restricts global generalizability. Second, a notable aspect of our methodology was using SMOTE to address the severe class imbalance. While this significantly enhanced sensitivity (0.800), ensuring fewer high-risk patients are missed, it contributed to a trade-off in precision (PPV = 0.500). However, for life-threatening complications like sepsis, prioritizing high sensitivity is clinically preferred. Third, while LASSO and SVM-RFE performed effectively, future studies could explore other feature selection algorithms, such as Boruta, to further optimize the predictor subset. Fourth, due to the reliance on retrospective EMR data, we primarily utilized phenotypic susceptibility results; the lack of detailed genotypic resistance mechanisms (e.g., specific carbapenemase production) prevented a deeper molecular analysis of sepsis progression. Finally, our validation was spatial rather than temporal, and the model relies on static baseline measurements without incorporating dynamic time-series data. Future prospective, multicenter studies incorporating dynamic trajectory analysis and genomic data are warranted to evaluate the real-world clinical utility of this tool (Fleuren et al., 2020).

In conclusion, we have developed and validated a pathogen-specific, interpretable machine learning model for predicting sepsis in MDR-PA infections. By identifying high-risk patients through readily available clinical parameters, our tool addresses the limitations of generic scoring systems (Kijpaisalratana et al., 2022). The integration of this model into clinical practice, facilitated by our web-based calculator, holds promise for optimizing resource allocation and improving outcomes in the growing battle against antimicrobial resistance.

## 5 Conclusions

We developed and externally validated an interpretable machine learning model for predicting sepsis in patients with MDR-PA infections. Using six routine predictors: invasive catheters, prior antibiotic exposure, calcium, COPD, RDW-SD, and intra-abdominal infection, the Random Forest model outperformed traditional scores like SOFA and SAPS II. By incorporating SHAP analysis and a web-based calculator, this study provides a transparent, practical tool to support early risk stratification and timely clinical intervention for this high-risk population.

## Data Availability

The original contributions presented in the study are included in the article/**Supplementary Material**. Further inquiries can be directed to the corresponding authors.

## References

[B1] Bova R. Griggio G. Vallicelli C. Santandrea G. Coccolini F. Ansaloni L. . (2024). Source control and antibiotics in intra-abdominal infections. Antibiotics (Basel) 13 (8), 776. doi: 10.3390/antibiotics13080776. PMID: 39200076 PMC11352101

[B2] Cilloniz C. Ward L. Mogensen M. L. Pericàs J. M. Méndez R. Gabarrús A. . (2023). Machine-learning model for mortality prediction in patients with community-acquired pneumonia: Development and validation study. Chest 163, 77–88. doi: 10.1016/j.chest.2022.07.005. PMID: 35850287

[B3] Cui X. D. Liu S. B. Wang R. Y. He D. D. Pan Y. S. Yuan L. . (2025). Investigation on the reversal effect of closantel on colistin resistance in MCR-1 positive Escherichia coli based on dose-response relationship. J. Antimicrob. Chemother. 80, 528–537. doi: 10.1093/jac/dkae441. PMID: 39658100

[B4] Desautels T. Calvert J. Hoffman J. Jay M. Kerem Y. Shieh L. . (2016). Prediction of sepsis in the intensive care unit with minimal electronic health record data: A machine learning approach. JMIR Med. Inform. 4, e28. doi: 10.2196/medinform.5909. PMID: 27694098 PMC5065680

[B5] Fernandes C. Pereira L. (2024). Hypocalcemia in critical care settings, from its clinical relevance to its treatment: A narrative review. Anaesth. Crit. Care Pain Med. 43, 101438. doi: 10.1016/j.accpm.2024.101438. PMID: 39395659

[B6] Fernando S. M. Tran A. Taljaard M. Cheng W. Rochwerg B. Seely A. J. E. . (2018). Prognostic accuracy of the quick sequential organ failure assessment for mortality in patients with suspected infection: A systematic review and meta-analysis. Ann. Intern. Med. 168, 266–275. doi: 10.7326/m17-2820. PMID: 29404582

[B7] Fleuren L. M. Klausch T. L. T. Zwager C. L. Schoonmade L. J. Guo T. Roggeveen L. F. . (2020). Machine learning for the prediction of sepsis: A systematic review and meta-analysis of diagnostic test accuracy. Intensive Care Med. 46, 383–400. doi: 10.1007/s00134-019-05872-y. PMID: 31965266 PMC7067741

[B8] Ghassemi M. Oakden-Rayner L. Beam A. L. (2021). The false hope of current approaches to explainable artificial intelligence in health care. Lancet Digit Health 3, e745–e750. doi: 10.1016/s2589-7500(21)00208-9. PMID: 34711379

[B9] Grießhammer A. de la Cuesta-Zuluaga J. Müller P. Gekeler C. Homolak J. Chang H. . (2025). Non-antibiotics disrupt colonization resistance against enteropathogens. Nature 644, 497–505. doi: 10.1038/s41586-025-09217-2. PMID: 40670795 PMC12350171

[B10] Herrera S. Bodro M. Soriano A. (2021). Predictors of multidrug resistant Pseudomonas aeruginosa involvement in bloodstream infections. Curr. Opin. Infect. Dis. 34, 686–692. doi: 10.1097/qco.0000000000000768. PMID: 34310454

[B11] Hsu C. L. Wu P. C. Wu F. Z. Yu H. C. (2024). LASSO-derived model for the prediction of lean-non-alcoholic fatty liver disease in examinees attending a routine health check-up. Ann. Med. 56, 2317348. doi: 10.1080/07853890.2024.2317348. PMID: 38364216 PMC10878349

[B12] Hsu W. H. Ko A. T. Weng C. S. Chang C. L. Jan Y. T. Lin J. B. . (2023). Explainable machine learning model for predicting skeletal muscle loss during surgery and adjuvant chemotherapy in ovarian cancer. J. Cachexia Sarcopenia Muscle 14, 2044–2053. doi: 10.1002/jcsm.13282. PMID: 37435785 PMC10570082

[B13] Hunter C. J. Marhoffer E. A. Holleck J. L. Ein Alshaeba S. Grimshaw A. A. Chou A. . (2025). Effect of empiric antibiotics against Pseudomonas aeruginosa on mortality in hospitalized patients: A systematic review and meta-analysis. J. Antimicrob. Chemother. 80, 322–333. doi: 10.1093/jac/dkae422. PMID: 39656468

[B14] Hurley J. (2025). Associations between Candida and Staphylococcus aureus, Pseudomonas aeruginosa, and Acinetobacter species as ventilator-associated pneumonia isolates in 84 cohorts of ICU patients. Microorganisms 13 (6), 1181. doi: 10.3390/microorganisms13061181. PMID: 40572069 PMC12194939

[B15] Kijpaisalratana N. Sanglertsinlapachai D. Techaratsami S. Musikatavorn K. Saoraya J. (2022). Machine learning algorithms for early sepsis detection in the emergency department: A retrospective study. Int. J. Med. Inform. 160, 104689. doi: 10.1016/j.ijmedinf.2022.104689. PMID: 35078027

[B16] Kulkarni P. A. Singh H. (2023). Artificial intelligence in clinical diagnosis: Opportunities, challenges, and hype. Jama 330, 317–318. doi: 10.1001/jama.2023.11440. PMID: 37410477

[B17] Kunz Coyne A. J. El Ghali A. Holger D. Rebold N. Rybak M. J. (2022). Therapeutic strategies for emerging multidrug-resistant Pseudomonas aeruginosa. Infect. Dis. Ther. 11, 661–682. doi: 10.1007/s40121-022-00591-2. PMID: 35150435 PMC8960490

[B18] Lauritsen S. M. Kristensen M. Olsen M. V. Larsen M. S. Lauritsen K. M. Jørgensen M. J. . (2020). Explainable artificial intelligence model to predict acute critical illness from electronic health records. Nat. Commun. 11, 3852. doi: 10.1038/s41467-020-17431-x. PMID: 32737308 PMC7395744

[B19] Martin-Loeches I. Antonelli M. Cuenca-Estrella M. Dimopoulos G. Einav S. De Waele J. J. . (2019). ESICM/ESCMID task force on practical management of invasive candidiasis in critically ill patients. Intensive Care Med. 45, 789–805. doi: 10.1007/s00134-019-05599-w. PMID: 30911804

[B20] Minasi A. Andreadi A. Maiorino A. Giudice L. De Taddeo S. D'Ippolito I. . (2023). Hypocalcemia is associated with adverse outcomes in patients hospitalized with COVID-19. Endocrine 79, 577–586. doi: 10.1007/s12020-022-03239-w. PMID: 36350462 PMC9643940

[B21] Montrucchio G. Balzani E. Sales G. Vaninetti A. Grillo F. Trompeo A. C. . (2024). Multidrug-resistant pathogens and ventilator-associated pneumonia in critically ill COVID-19 and non-COVID-19 patients: A prospective observational monocentric comparative study. Respir. Res. 25, 168. doi: 10.1186/s12931-024-02779-1. PMID: 38637766 PMC11027225

[B22] Moor M. Rieck B. Horn M. Jutzeler C. R. Borgwardt K. (2021). Early prediction of sepsis in the ICU using machine learning: A systematic review. Front. Med. (Lausanne) 8. doi: 10.3389/fmed.2021.607952. PMID: 34124082 PMC8193357

[B23] Opron K. Begley L. A. Erb-Downward J. R. Li G. Alexis N. E. Barjaktarevic I. . (2024). Loss of airway phylogenetic diversity is associated with clinical and pathobiological markers of disease development in chronic obstructive pulmonary disease. Am. J. Respir. Crit. Care Med. 210, 186–200. doi: 10.1164/rccm.202303-0489OC. PMID: 38261629 PMC11273318

[B24] Pitiriga V. C. Bakalis J. Campos E. Kanellopoulos P. Sagris K. Saroglou G. . (2024). Central venous catheters versus peripherally inserted central catheters: A comparison of indwelling time resulting in colonization by multidrug-resistant pathogens. Antibiotics (Basel) 13. doi: 10.3390/antibiotics13010089. PMID: 38247648 PMC10812679

[B25] Prasad P. A. Fang M. C. Abe-Jones Y. Calfee C. S. Matthay M. A. Kangelaris K. N. (2020). Time to recognition of sepsis in the emergency department using electronic health record data: A comparative analysis of systemic inflammatory response syndrome, sequential organ failure assessment, and quick sequential organ failure assessment. Crit. Care Med. 48, 200–209. doi: 10.1097/ccm.0000000000004132. PMID: 31939788 PMC7494056

[B26] Rani N. D. Babu M. (2024). Improved rank-based recursive feature elimination method based ovarian cancer detection model via customized deep architecture. Comput. Methods Programs Biomed. 256, 108358. doi: 10.1016/j.cmpb.2024.108358. PMID: 39191100

[B27] Routsi C. Gkoufa A. Arvaniti K. Kokkoris S. Tourtoglou A. Theodorou V. . (2020). De-escalation of antimicrobial therapy in ICU settings with high prevalence of multidrug-resistant bacteria: A multicentre prospective observational cohort study in patients with sepsis or septic shock. J. Antimicrob. Chemother. 75, 3665–3674. doi: 10.1093/jac/dkaa375. PMID: 32865203

[B28] Rudin C. (2019). Stop explaining black box machine learning models for high stakes decisions and use interpretable models instead. Nat. Mach. Intell. 1, 206–215. doi: 10.1038/s42256-019-0048-x. PMID: 35603010 PMC9122117

[B29] Sati H. Carrara E. Savoldi A. Hansen P. Garlasco J. Campagnaro E. . (2025). The WHO bacterial priority pathogens list 2024: A prioritisation study to guide research, development, and public health strategies against antimicrobial resistance. Lancet Infect. Dis. 25, 1033–1043. doi: 10.1016/s1473-3099(25)00118-5. PMID: 40245910 PMC12367593

[B30] Sendak M. P. Gao M. Brajer N. Balu S. (2020a). Presenting machine learning model information to clinical end users with model facts labels. NPJ Digit Med. 3, 41. doi: 10.1038/s41746-020-0253-3. PMID: 32219182 PMC7090057

[B31] Sendak M. P. Ratliff W. Sarro D. Alderton E. Futoma J. Gao M. . (2020b). Real-world integration of a sepsis deep learning technology into routine clinical care: Implementation study. JMIR Med. Inform. 8, e15182. doi: 10.2196/15182. PMID: 32673244 PMC7391165

[B32] Serafim R. Gomes J. A. Salluh J. Póvoa P. (2018). A comparison of the quick-SOFA and systemic inflammatory response syndrome criteria for the diagnosis of sepsis and prediction of mortality: A systematic review and meta-analysis. Chest 153, 646–655. doi: 10.1016/j.chest.2017.12.015. PMID: 29289687

[B33] Shan W. Sun D. Liu Z. (2024). “ Predicting sepsis onset in ICU patients using machine learning and feature section: A case study of MIMIC-IV data,” in 2024 IEEE International Conference on Medical Artificial Intelligence (MedAI). (New York, NY: IEEE). 546–551.

[B34] Shi X. Nikolic G. Epelde G. Arrúe M. Bidaurrazaga Van-Dierdonck J. Bilbao R. . (2021). An ensemble-based feature selection framework to select risk factors of childhood obesity for policy decision making. BMC Med. Inform. Decis. Mak 21, 222. doi: 10.1186/s12911-021-01580-0. PMID: 34289843 PMC8293582

[B35] Silva M. A. Hamilton E. J. Russell D. A. Game F. Wang S. C. Baptista S. . (2025). Diabetic foot ulcer classification models using artificial intelligence and machine learning techniques: Systematic review. J. Med. Internet Res. 27, e69408. doi: 10.2196/69408. PMID: 40991939 PMC12508669

[B36] Singer M. Deutschman C. S. Seymour C. W. Shankar-Hari M. Annane D. Bauer M. . (2016). The third international consensus definitions for sepsis and septic shock (Sepsis-3). Jama 315, 801–810. doi: 10.1001/jama.2016.0287. PMID: 26903338 PMC4968574

[B37] Swanson K. Wu E. Zhang A. Alizadeh A. A. Zou J. (2023). From patterns to patients: Advances in clinical machine learning for cancer diagnosis, prognosis, and treatment. Cell 186, 1772–1791. doi: 10.1016/j.cell.2023.01.035. PMID: 36905928

[B38] Thelagathoti R. K. Tom W. A. Chandel D. S. Jiang C. Krzyzanowski G. Olou A. . (2025). A hybrid sequential feature selection approach for identifying new potential mRNA biomarkers for Usher syndrome using machine learning. Biomolecules 15 (7), 963. doi: 10.3390/biom15070963. PMID: 40723835 PMC12293090

[B39] Tuon F. F. Dantas L. R. Suss P. H. Tasca Ribeiro V. S. (2022). Pathogenesis of the Pseudomonas aeruginosa biofilm: A review. Pathogens 11. doi: 10.3390/pathogens11030300. PMID: 35335624 PMC8950561

[B40] Vena A. Giacobbe D. R. Castaldo N. Cattelan A. Mussini C. Luzzati R. . (2020). Clinical experience with ceftazidime-avibactam for the treatment of infections due to multidrug-resistant gram-negative bacteria other than carbapenem-resistant Enterobacterales. Antibiotics (Basel) 9 (2), 71. doi: 10.3390/antibiotics9020071. PMID: 32050434 PMC7168189

[B41] Vena A. Schenone M. Corcione S. Giannella M. Pascale R. Giacobbe D. R. . (2024). Impact of adequate empirical combination therapy on mortality in septic shock due to Pseudomonas aeruginosa bloodstream infections: A multicentre retrospective cohort study. J. Antimicrob. Chemother. 79, 2846–2853. doi: 10.1093/jac/dkae296. PMID: 39224938

[B42] Yan D. Xie X. Fu X. Pei S. Wang Y. Deng Y. . (2023). U-shaped association between serum calcium levels and 28-day mortality in patients with sepsis: A retrospective analysis of the MIMIC-III database. Shock 60, 525–533. doi: 10.1097/shk.0000000000002203. PMID: 37566809 PMC10581423

[B43] Zhang P. Guo Q. Wei Z. Yang Q. Guo Z. Shen L. . (2021). Baicalin represses type three secretion system of Pseudomonas aeruginosa through PQS system. Molecules 26 (6). doi: 10.3390/molecules26061497. PMID: 33801847 PMC8001617

[B44] Zoccali C. Mallamaci F. (2023). Innate immunity system in patients with cardiovascular and kidney disease. Circ. Res. 132, 915–932. doi: 10.1161/circresaha.122.321749. PMID: 37053283

