# Supplementary material for: Machine learning-based risk prediction model for sepsis development in patients with multidrug-resistant Pseudomonas aeruginosa infections: a multicenter retrospective cohort study
Source: Front Cell Infect Microbiol. 2026 Apr 10;16:1792743. doi: 10.3389/fcimb.2026.1792743 (PMC13105939; doi:10.3389/fcimb.2026.1792743)
Supplement: Supplementary file 1 [file DataSheet1.docx]

**Supplementary Figure 1** Study Timeline and Window Definitions

**
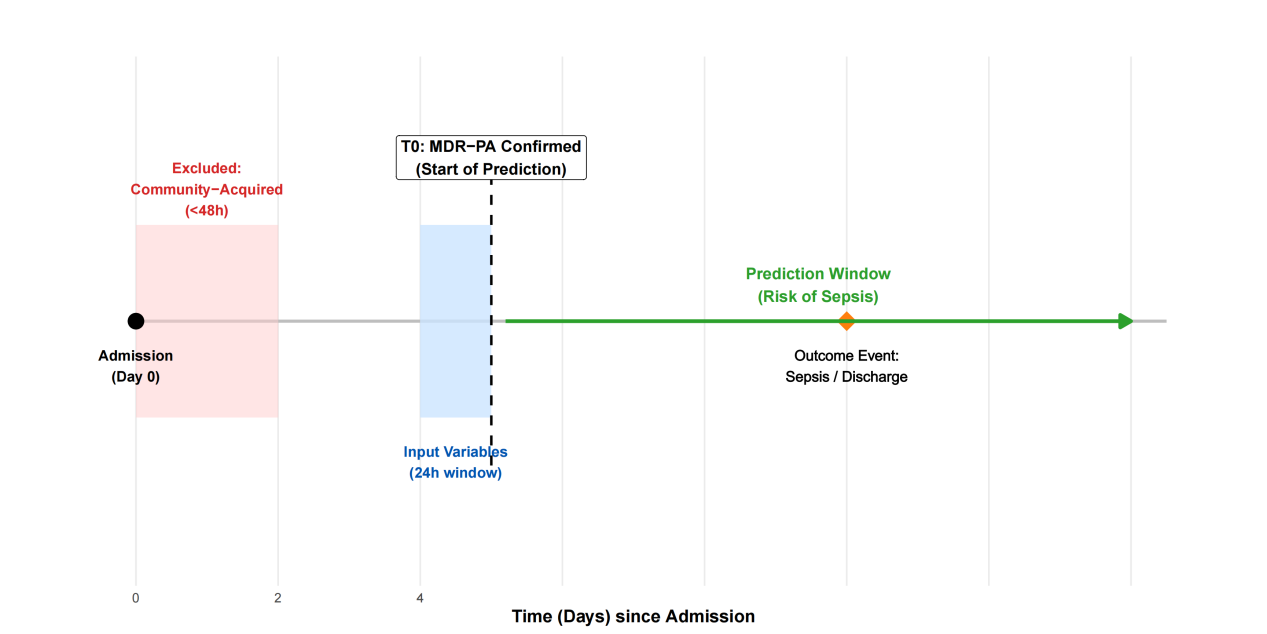
**

MDR-PA: multidrug-resistant *Pseudomonas aeruginosa*.

**Supplementary Figure 2** ROC curve of the RF model based on MICE and comeplete case analysis in internal test. (A. MICE case; B. Comeplete case).


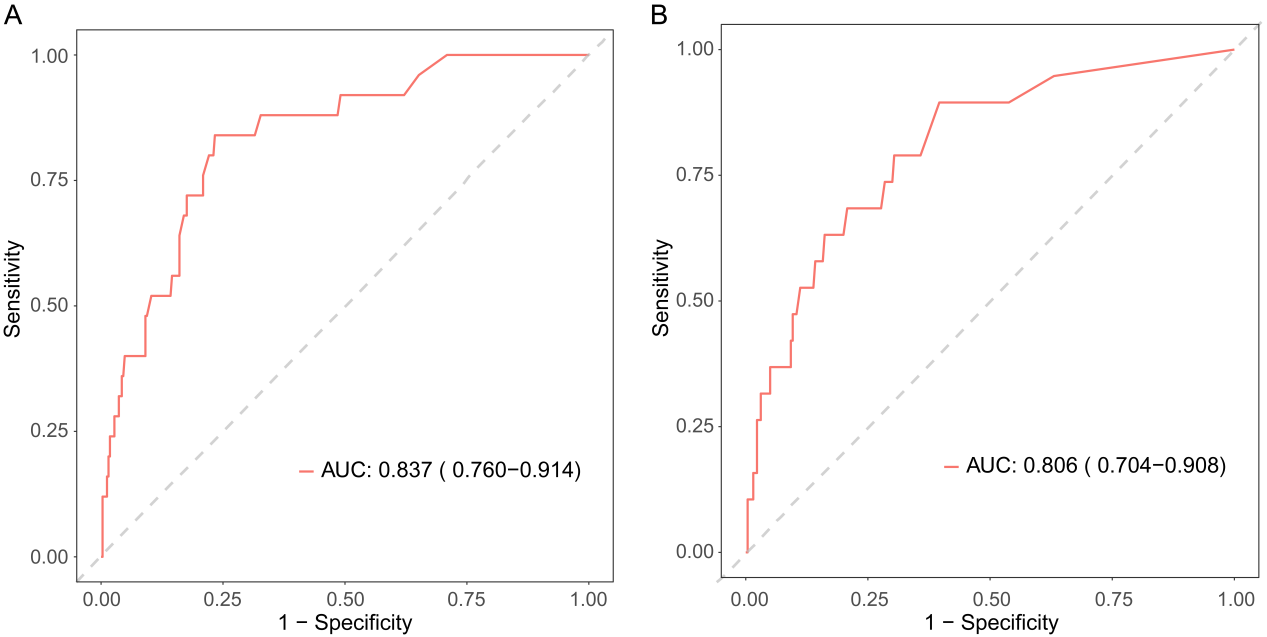


AUC: Area Under Curve; RF: Random Forest; MICE: Multivariate Imputation by Chained Equations.

**Supplementary Figure 3** ROC curve of the RF model based on MICE and comeplete case analysis in external test. (A. MICE case; B. Comeplete case).


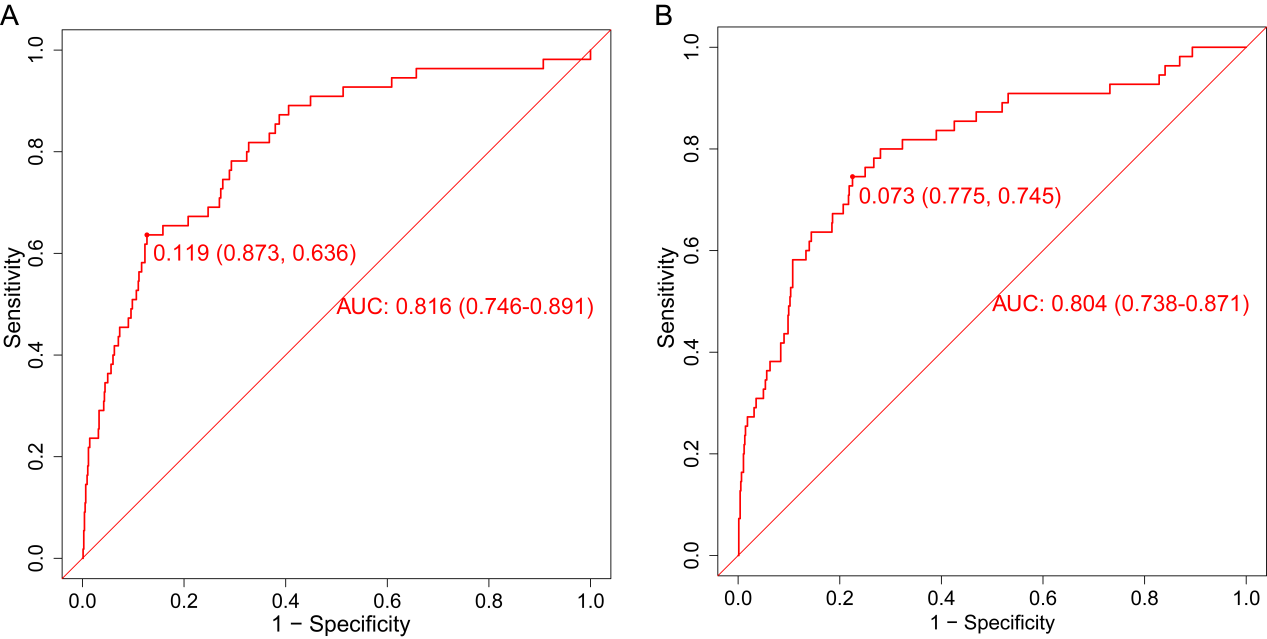


AUC: Area Under Curve; RF: Random Forest; MICE: Multivariate Imputation by Chained Equations.
